# 2D Titanium Carbide MXene and Single‐Molecule Fluorescence: Distance‐Dependent Nonradiative Energy Transfer and Leaflet‐Resolved Dye Sensing in Lipid Bilayers

**DOI:** 10.1002/adma.202411724

**Published:** 2024-10-24

**Authors:** Lorena Manzanares, Dahnan Spurling, Alan M. Szalai, Tim Schröder, Ece Büber, Giovanni Ferrari, Martin R. J. Dagleish, Valeria Nicolosi, Philip Tinnefeld

**Affiliations:** ^1^ Univ. Lille CNRS Centrale Lille Univ. Polytechnique Hauts‐de‐France UMR 8520 – IEMN – Institut d'Electronique de Microélectronique et de Nanotechnologie Lille F‐59000 France; ^2^ Department of Chemistry and Center for NanoScience Ludwig‐Maximilians‐University Butenandtstraße 5–13 81377 Munich Germany; ^3^ School of Chemistry, Centre for Research on Adaptive Nanostructures and Nanodevices (CRANN) & Advanced Materials Bio‐Engineering Research Centre (AMBER) Trinity College Dublin Dublin 2 Ireland; ^4^ Centro de Investigaciones en Bionanociencias (CIBION), Consejo Nacional de Investigaciones Científicas y Técnicas (CONICET) Godoy Cruz 2390 Ciudad Autónoma de Buenos Aires C1425FQD Argentina

**Keywords:** 2D materials, DNA origami, energy transfer, fluorescence microscopy, MXene, single‐molecule, supported lipid bilayers

## Abstract

Despite their growing popularity, many fundamental properties and applications of MXene materials remain underexplored. Here, the nonradiative energy transfer properties of 2D titanium carbide MXene are investigated and their application in single‐molecule biosensing is explored for the first time. DNA origami positioners are used for single dye placement immobilized by a specific chemistry based on glycine‐MXene interactions, allowing precise control of their orientation on the surface. Each DNA origami structure carries a single dye molecule at predetermined heights. Single‐molecule fluorescence confocal microscopy reveals that energy transfer of an organic emitter (ATTO 542) on transparent thin films made of spincast Ti_3_C_2_T*_x_* flakes follows a cubic distance dependence, where 50% of energy transfer efficiency is reached at 2.7 nm (*d*_0_). MXenes are applied as short‐distance spectroscopic nanorulers, determining *z* distances of dye‐labeled supported lipid bilayers fused on MXene's hydrophilic surface. Hydration layer (2.1 nm) and lipid bilayer thickness (4.5 nm) values that agree with the literature are obtained. These results highlight titanium carbide MXenes as promising substrates for single‐molecule biosensing of ultrathin assemblies, owing to their sensitivity near the interface, a distance regime that is typically inaccessible to other energy transfer tools.

## Graphical abstract

MXenes' distance‐dependent energy transfer properties are examined using single‐molecule fluorescence microscopy and DNA origami nanopositioners. The findings reveal MXenes as short‐distance spectroscopic nanorulers, achieving 50% energy transfer efficiency at 2.7 nm with a cubic power law dependency. Single‐molecule leaflet‐resolved dye sensing of lipid bilayers are demonstrated with MXene energy transfer due to MXenes’ sensitivity at the interface.

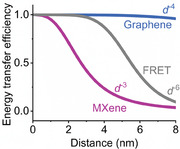

## 1 Introduction

The phenomenon of non‐radiative energy transfer (ET) from a donor to an acceptor system has been well‐understood for decades.^[^ ^1^, ^2^, ^3^ ^]^ The process is strongly distance‐dependent following a near field interaction and its efficiency is contingent on several factors, including spectral overlap between the donor's emission and the acceptor's absorption, screening of the electric field of the donor transition‐dipole‐moment (TDM) in the surrounding medium, and the dimensionality of the donor and acceptor.

A well‐known example of distance‐dependent non‐radiative energy transfer is Förster resonance energy transfer or FRET,^[^ ^3^ ^]^ where a donor dye in the excited state transfers its energy to an acceptor dye in the ground state. Biomolecules can be labeled with a donor‐acceptor pair and the *d* ^−6^ distance dependence of the energy transfer in the 3–10 nm range is typically used to monitor conformational changes, dynamics, and binding. However, measuring absolute distances remains challenging due to the nontrivial dependencies on the relative orientations of donor and acceptor molecules, which make the analysis and interpretation of FRET data intricate.^[^ ^4^, ^5^, ^6^, ^7^, ^8^ ^]^

In surface‐induced ET, only one dye label (donor) is required and substrates like metal films and graphene act as dark, broadband and unbleachable energy acceptors, eliminating issues such as bleed‐through of donor emission, direct acceptor excitation, and problems arising from acceptor photophysics.^[^ ^4^, ^5^, ^6^, ^8^ ^]^ For many years, the interest in surface‐induced ET has been merely fundamental, yet it has recently attracted interest for applications in single‐molecule fluorescence microscopy and spectroscopy.^[^ ^9^, ^10^ ^]^ The dependence between the fluorescence lifetime or intensity of an emitter and the distance from the acceptor surface has been utilized to map different biological nano‐environments by localizing fluorescently labeled molecules positioned near these surfaces and converting their fluorescence intensity or lifetime values into nanometer distances.^[^ ^11^ ^]^ Different surfaces provide a range of sensitivities at different distance ranges in the *z* or axial direction that extent beyond^[^ ^11^ ^]^ or below^[^ ^12^ ^]^ the FRET range. Monolayer graphene has shown an improved localization precision compared to gold, at the expense of a shorter dynamic range (up to 40 nm compared to 150 nm), and quartic distance dependency of the energy transfer efficiency, rapidly becoming a part of the single‐molecule research toolbox with applications in single‐molecule biosensing, biophysics and super‐resolution microscopy.^[^ ^14^, ^15^ ^]^ For instance, gold is used to probe large structures such as fixed cells^[^ ^10^, ^13^ ^]^ whereas graphene allows us to elucidate smaller structures, such as outer membrane protein complexes,^[^ ^14^ ^]^ DNA dynamics^[^ ^15^ ^]^ and DNA‐protein structural insights.^[^ ^16^ ^]^ The axial localization of dyes linked to supported lipid bilayers (SLBs), common biomembrane models, represents another key application of graphene as energy acceptor. Several ensemble‐level studies have been conducted with SLBs on graphene‐silica systems, revealing thickness variations and other biophysical features.^[^ ^4^, ^17^, ^18^ ^]^

MXenes – transition metal carbides, nitrides and carbonitrides with the formula M_*n*+1_X_*n*_T_*x*_ (where M denotes an early transition metal, X can be either carbon or nitrogen, T_*x*_ represents surface terminations, and the value of *n* ranges from 1 to 4) – are a large family of 2D materials discovered in 2011^[^ ^19^ ^]^ that have become important in the fields of energy storage,^[^ ^20^ ^]^ sensing^[^ ^21^ ^]^ and electromagnetic shielding.^[^ ^22^ ^]^ However, many of their fundamental properties including nonradiative ET remain unknown, relevant for both fundamental reasons and applications in optoelectronics^[^ ^23^, ^24^ ^]^ and single‐molecule biosensing.^[^ ^15^ ^]^ In the last few years, we have recognized the potential of low‐dimensional, metal‐like materials in enhancing the resolution of single‐molecule and super‐resolution fluorescence microscopy methods.^[^ ^15^, ^25^, ^26^ ^]^ MXenes have previously been reported to quench the fluorescence of adsorbed dyes^[^ ^27^, ^28^, ^29^ ^]^ but a quantitative understanding of this phenomenon and its scaling with distance is lacking. To investigate this, precise nanometer‐scale surface control is imperative. Our group has employed DNA nanotechnology coupled with single‐molecule fluorescence microscopy to achieve the controlled placement of fluorescent dyes on surfaces.^[^ ^30^, ^31^, ^32^, ^33^, ^34^ ^]^ Prior work on graphene‐mediated ET using DNA origami nanopositioners resulted in experimental energy transfer quantities that lie very close to theoretical considerations.^[^ ^34^ ^]^ We believe this is because on one hand, the addressability of the DNA origami technique allows us to control the placement of single fluorescent dyes with very high distance‐precision to the surface, that of a single nucleotide (0.34 nm). On the other hand, the dye molecules are covalently attached to the phosphate backbone of DNA via flexible linkers, enabling free rotation of the dye molecules in solution. As a result, single emitters can adopt multiple TDM orientations in DNA origami nanopositioners,^[^ ^33^ ^]^ important for distance calibrations, which are often modelled assuming rapidly rotating and flexible TDM orientations.^[^ ^11^ ^]^ And finally, DNA origami structures are handled in aqueous conditions, compatible with the biological applications that many of the distance calibrations will be used for.

We here describe a single‐molecule fluorescence microscopy study of MXene‐induced energy transfer using DNA origami nanopositioners on thin Ti_3_C_2_T*_x_* flakes − the most studied member of this family of 2D materials − spincast on glass coverslips. We investigate the distance dependence of the quenching phenomenon with DNA nanostructures carrying a single dye (ATTO 542) at controlled distances. In the absence of established surface immobilization chemistries for biomolecules on MXene materials, we developed a one‐step method based on the known glycine‐MXene interaction^[^ ^35^ ^]^ to control the specific placement of DNA origami on MXene flakes, through triglycine‐modified DNA strands. We demonstrated an application in sensing dye‐labeled supported lipid bilayers fused on the hydrophilic surface of titanium carbide MXene. Using MXene‐induced energy transfer (ET), we were able to determine hydration and bilayer thickness values at the single‐molecule level.

## 2 Results and Discussion

### 2.1 Thin Films of Ti_3_C_2_T*_x_* Flakes

**Figure 1:**
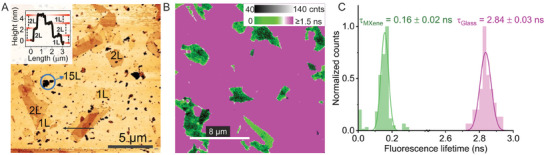
MXene films: Characterization with atomic force microscopy (AFM) and fluorescence lifetime imaging microscopy (FLIM) of film thickness variations and their effect on ATTO 542′s fluorescence intensity and lifetime: A) Representative AFM image of MXene spincast films on glass (1L: one layer). Small multilayer structures are encircled. Inset: height profile of self‐folded flake. B) Representative FLIM image (20 × 20 µm, 100 nm pixel size) of flakes stained with 1 µm of ssDNA‐ATTO 542 in TAE‐Mg^+2^ buffer. A color‐coded gradient bar is shown for fast lifetime values and a grey scale gradient for intensity values. Green areas: quenched fluorescence (MXene flakes); magenta areas: unquenched (glass). Light to dark green areas are indicative of MXene thickness variations. C) Fluorescence lifetime distribution of multiple 500 × 500 nm areas (*n* = 179, green) sampled from different MXene layers and from glass areas (*n =* 50, pink). Lifetime values were calculated via reconvolution fitting with the measured instrument response function (IRF). The distributions were fitted using a Gaussian model. The average lifetime value and standard deviation of the distributions are displayed.

We used Ti_3_C_2_T*_x_* flakes obtained by LiF‐HCl exfoliation of Ti_3_AlC_2_ and spincast on glass coverslips (see Methods in Supporting Information). We obtained a discontinuous, optically transparent film (≈90% transmittance).^[^ ^36^ ^]^ **Figure** **1**A shows atomic force micrographs of the films, depicting monolayer flakes that fold on themselves forming up to four‐layered regions with up to 4 µm of lateral size (see height profile in Inlet; 1 layer ≈1‐1.5 nm). Overall, the films consist of large flakes (a few microns of lateral size) from 1 to 5 layers co‐existing with smaller multilayer flakes (up to 40 layers, ≤500 nm of lateral size). Supplementary Figure S1 (Supporting Information) shows the typical UV‐Vis absorption spectrum of a Ti_3_C_2_T*_x_* aqueous dispersion, showing the characteristic peak at ≈760 nm. The MXene films do not exhibit autofluorescence (Figure S1, Supporting Information, inset). Figure S2 (Supporting Information) shows a single Ti_3_C_2_T*_x_* flake imaged by scanning electron microscopy (SEM) and its elemental composition obtained with energy‐dispersive X‐ray (EDX) spectroscopy. The X‐ray diffraction (XRD) patterns of the dry spincast films show the typical (00l) and (002) reflections of MXenes (Figure S3‐A, Supporting Information). The latter (7.14°) gave a *d*‐spacing of 1.24 nm and an interlayer spacing of 0.3 nm, values that are in good agreement with those reported for films of exfoliated Ti_3_C_2_T*_x_* MXene.^[^ ^37^ ^]^ When the films were wetted in a thin electrolyte layer containing Na^+^ or Mg^2+^ ions (resembling the conditions used in the DNA‐MXene interfaces for the energy transfer studies described next), the (002) reflection peaks shifted to 5.69° and 5.45°, respectively (Figure S3‐B, Supporting Information). This resulted in increased *d*‐spacing (and interlayer spacing) values, that is, 1.55 (0.61 nm) and 1.62 nm (0.68 nm), due to spontaneously intercalated Na^+^ and Mg^2+^ ions, respectively. The difference between these values is in agreement with the larger number of coordinated water molecules in the hydration shell of Mg^2+^ in comparison to that of Na^+^.^[^ ^38^, ^39^ ^]^ Overall, the characterization data align with the typical features of Ti_3_C_2_T*_x_* MXene.

We performed a staining experiment of the MXene flakes with fluorescent dyes^[^ ^40^ ^]^ to assess whether drastic differences occur in the fluorescence lifetime of the dye marked by the number of MXene layers. Single stranded DNA (ssDNA) end‐labeled with ATTO 542 was used to create a fluorescent film on the MXene‐on‐glass substrates. Figure S1 (Supporting Information) depicts the spectral overlap of the ATTO 542′s emission spectrum with the absorbance spectrum of a MXene dispersion. We chose a dye that overlaps with the flat region of MXene's absorption spectrum to focus on nonradiative energy transfer, avoiding possible convoluted effects on nonradiative and radiative decay rates near the 760‐nm‐centered absorption peak, the nature of which remains unclear.^[^ ^41^, ^42^ ^]^ Figure 1B shows a fluorescence lifetime imaging microscopy (FLIM) scan of “stained” flakes with ATTO 542‐ssDNA, where the darkest area is assumed to be the thickest MXene layers due to increased absorption in those regions. Figure 1C shows the narrow lifetime distributions of 179 areas (0.25 µm^2^) across flakes of different thicknesses – having a similar width to the unquenched dye film on glass areas. There appears to be a negligible effect of MXene thickness on the fluorescence lifetime of ATTO 542 dyes (τ_ATTO542_), which lies in contrast to graphene, where the emitter's decay rate has been shown to be proportional to the number of layers.^[^ ^43^, ^44^ ^]^ The thickness‐independent behavior of MXenes has been previously observed for other spectroscopic properties, ascribed to their unusual weak interlayer coupling.^[^ ^42^, ^45^, ^46^ ^]^ In our case, the short quenching dynamic range is thought to play a role, as it will be discussed later.

### 2.2 The DNA Origami–MXene Interface

**Scheme 1:**
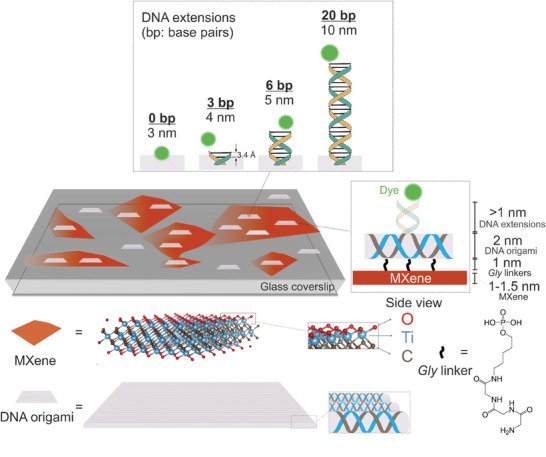
DNA origami‐MXene interface design for distance‐dependent energy transfer studies at the single‐molecule level. MXene flakes spincast on glass coverslips make up an optically transparent, discontinuous film with bare glass regions and flake regions. The surface groups ‐O, ‐OH, ‐F (only ‐O is depicted for the sake of simplicity) of MXenes render hydrophilic surfaces (shown in red). Rectangular DNA origami nanostructures (grey tiles) are designed to carry single donor dyes (green spheres) at different heights from the MXene surface, by modifications introduced to staple strands (grey). The long ssDNA scaffold strand is shown in blue. The contour lengths of the DNA extensions are calculated according to the number of protruding base pairs (DNA extensions, in blue and yellow), where 0, 3, 6 and 20 bp protrusions result in of 3, 4, 5 and 10 nm heights for dye placement. The DNA origami nanostructures are specifically immobilized via glycine (*Gly*) linkers (black lines) to the surface of MXenes and non‐specifically adsorbed on glass.

**Scheme** **1** shows a simplified representation of the system we used to probe the distance dependence of MXene‐induced ET. We chose a flat DNA origami nanostructure (known as new rectangular origami “NRO”)^[^ ^47^, ^48^ ^]^ to position the dye at controlled heights from the MXene surface. Figure S4 (Supporting Information) shows the caDNAno design of the NRO origami structures.

We began by establishing an immobilization chemistry to specifically anchor these nanostructures to the material surface. Inspired by the glycine‐MXene chemistry previously reported in energy storage works,^[^ ^35^ ^]^ we incorporated 6 internal staples end‐labeled with commercially available triglycine moieties (referred to as Gly linkers in Scheme 1) in the NRO structure, together with the dye molecule facing in the opposite direction (0 bp, 3 nm, shown in Scheme 1). Glycine has been reported to bind to MXenes by forming a N–Ti covalent bond via glycine's primary amine.^[^ ^35^, ^49^ ^]^ The triglycine molecule was estimated to add ca. 1 nm spacing distance from the surface (more details in Methods). A control experiment was designed with an NRO structure without Gly linkers to test the specificity of the immobilization chemistry (under Mg^2+^‐free conditions). Figure S5 (Supporting Information) shows the AFM characterization of NRO nanostructures adsorbed on mica under the buffer conditions (free of divalent salts) chosen for their specific immobilization on MXene, where most of the structures appear intact.

**Figure 2:**
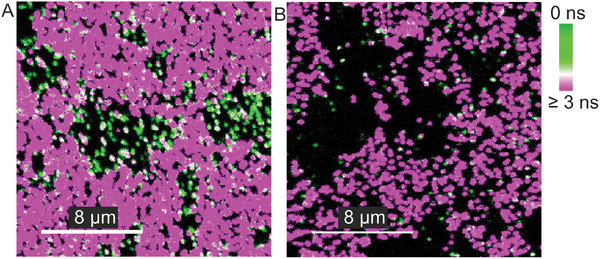
Specific immobilization of ATTO 542‐labeled DNA origami nanostructures (NROs) on MXene films: Fluorescence lifetime imaging microscopy (FLIM) scans (20 × 20 µm) of NROs A) with and B) without glycine (*Gly*) linkers (in PBS‐Na^+^ buffer) A color‐coded gradient bar is shown for fast lifetime values. Unquenched NROs adsorbed on glass are seen as magenta spots and quenched NROs immobilized on MXene flakes are seen as green spots.

**Figure** **2**A and B show FLIM maps of NROs with and without *Gly*, respectively. In the glycinated system, one can see quenched dye molecules located on MXene micron‐sized regions. The significantly reduced presence of NROs without *Gly* in MXene areas (i.e., tenfold less in a 25 µm^2^ area) with respect to glass areas, confirms that the immobilization on MXene takes place specifically via Gly. It is, however, possible to immobilize NROs without Gly in the presence of divalent salts (Figure S6, Supporting Information) but they are expected to land on either side forming Mg^2+^ bridges with the phosphate backbone of dsDNA onto the MXene surface.^[^ ^28^ ^]^

### 2.3 Distance Dependence of MXene‐Induced Energy Transfer

Having established an immobilization protocol for the DNA origami‐MXene interface, we designed four NRO nanopositioners granting a range of heights to the dye from the MXene surface (Scheme 1). The 3 nm distance was designed as previously described for the 0 bp nanopositioner. Double‐stranded DNA extensions were incorporated upwards to place the dye further away from the surface. The caDNAno designs are shown in Figure S4 (Supporting Information).

To determine the average orientation of dsDNA protruding from the NRO (20 bp), we measured the height of the donor dye with a monolayer of graphene as energy acceptor, which is a well‐calibrated system in our lab. Figure S7 (Supporting Information) shows the resulting lifetime distribution and FLIM images, suggesting that a dye protruding at a contour length of 10 nm has an effective distance of 8 nm, corresponding to an averaged tilt angle of 49°.

**Figure 3:**
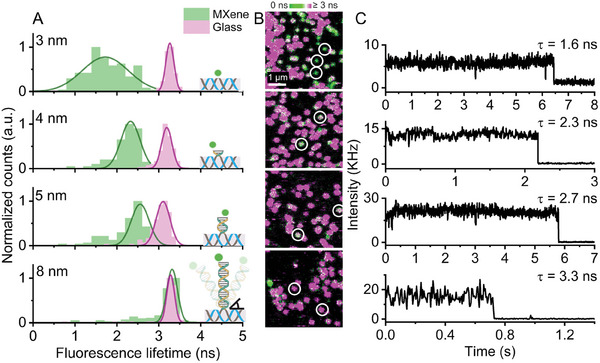
Single‐molecule derived data from ATTO 542 dyes placed at different heights from the MXene substrate through DNA origami nanopositioners (NROs): A) Lifetime distributions of *n* ≈ 150 molecules on MXene (green). Reference (glass) lifetime distributions are shown in pink. The schemes of the NRO nanopositioners used to probe distances between 3 and 8 nm to the MXene surface are also depicted. The distributions were fitted using a Gaussian model. B) Representative fluorescence lifetime imaging microscopy (FLIM) scans (4.5 × 4.5 µm) for each NRO with color‐coded fast lifetime values of molecules on MXene (varying colors) and glass (magenta spots). White circles are used to indicate correctly oriented NROs on MXenes. C) Representative single‐molecule intensity trajectories (showing one‐step photobleaching) and fluorescence lifetime values of ATTO 542 for each origami.

**Figure** **3**A shows the distributions of the fluorescence lifetime values derived from single‐molecule confocal microscopy measurements of the different nanopositioners on MXene. Reference measurements were carried out separately on BSA‐biotin‐NeutrAvidin‐coated glass with biotinylated NROs to extract the dyes’ reference lifetime values in the absence of MXene‐induced quenching.

The histograms show a clear lifetime shift as the dye‐MXene distance increases. The unquenched state is reached at ca. 8 nm, approximating the reference τ_ATTO542_ value of 3.3 ± 0.1 ns. The data dispersion at 3 nm, where nearly 50% of energy transfer is reached (known as the *d*_0_ value), is unavoidable as the function of the energy transfer rate shows the steepest gradient in this region.

For the 4 and 5 nm distances, the τ_ATTO542_ values of NROs on MXene were challenging to differentiate from glass adsorbed NROs at the point of data collection (Figure 3B). In order to simplify the analysis, the glass‐contributing regions were subtracted from the data (see Figure S8, Supporting Information for raw, untreated data). For the 8 nm distance, the color‐coded lifetimes of the dyes on MXene and glass could not be distinguished from each other during the measurements. Thus, the molecules were selected based on the lower density of NROs immobilized on MXene than that of glass.

**Figure 4:**
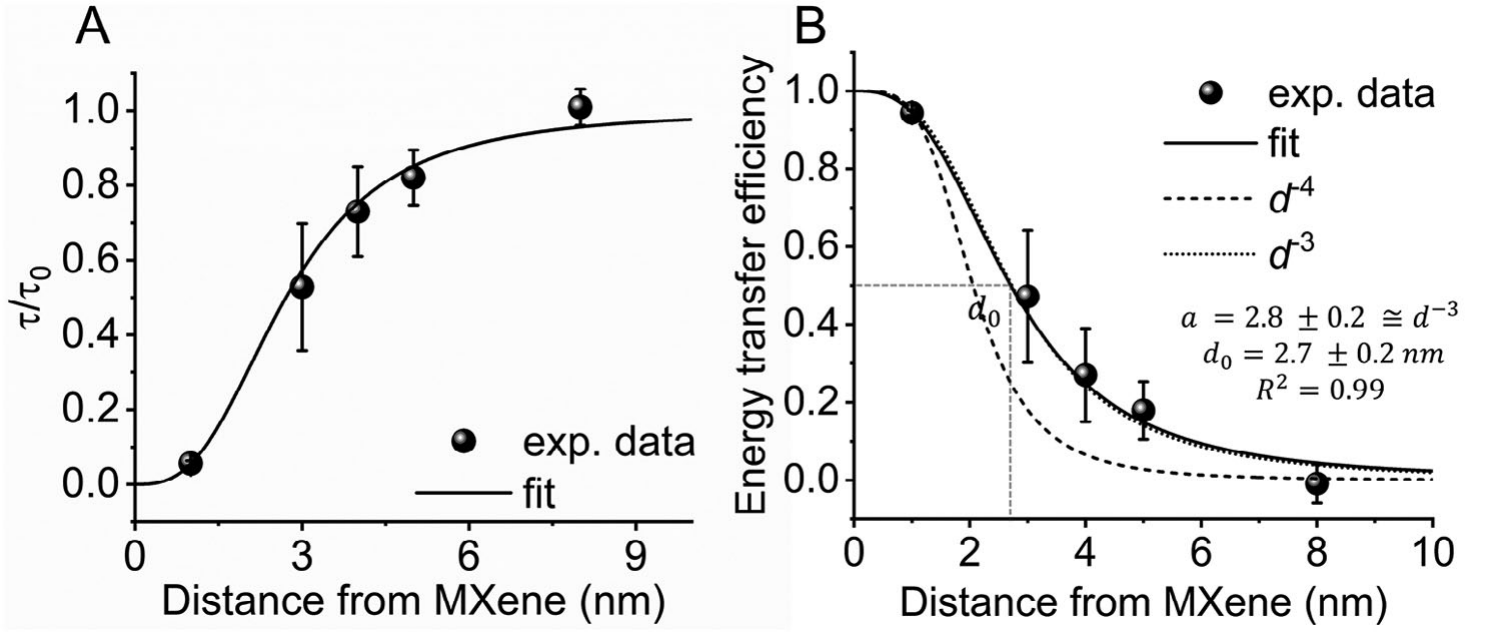
Distance dependence of MXene‐induced energy transfer: A) Normalized lifetime as a function of distance between ATTO 542 and the MXene substrate. B) Energy transfer efficiency calculated from fluorescence lifetime values as a function of the distance between ATTO 542 and MXene. Fitted curve of the energy transfer efficiency as the function of the distance *d* between MXene and emitter (R^2^ = 0.996), where *d*_0_ states the distance of 50% quenching efficiency, and from the fit equals 2.71 ± 0.16 nm. The parameter *a* stands for the power of distance dependency, which from the fit equals 2.84 ± 0.18. Standard deviation values in both plots were calculated using error propagation from τ/τ_0_ quantities. Values of τ/τ_0_ and ET_efficiency_ for 1 nm distance were calculated from ssDNA‐ATTO542 fluorescent films (ensemble experiments) and for 3 to 8 nm from DNA origami nanopositioners (single‐molecule level). Fitted curves for cubic (*d^−^* ^3^, dotted line) and quartic scaling laws (*d^−^* ^4^, dashed line) for bulk and surface processes, respectively, demonstrate adherence to *d^−^* ^3^.

Intensity traces in Figure 3C illustrating one‐step photobleaching are indicative of single molecules, showing that the analysis of fluorescence lifetimes for distances between 3 and 8 nm was carried out at the single‐molecule level. The lifetime values do not scale proportionally with the recorded intensity values due to the heterogeneous light absorption of the different MXene flake thicknesses, and other factors such as defocusing and sample drift effects. Thus, we calculated the energy transfer efficiency (ET) only from fluorescence lifetimes as it is inversely proportional to the sum of all depopulating rate constants of the excited state and is therefore not affected by the differences in the absorbance of mono‐ to multi‐layer MXene or sample drift. We plotted the normalized lifetime $\frac{\tau }{\tau_{0}}$, and the resulting ET efficiency values, calculated from $1-\frac{\tau }{\tau_{0}}$, against the distance from MXene surface in **Figure** **4**A,B, respectively, where τ is the fluorescence lifetime of ATTO 542 dyes on MXene flakes and τ_0_ is the fluorescence lifetime of the dye‐NROs on BSA‐biotin‐NeutrAvidin‐functionalized glass. An exception was made for the 1 nm distance. The lifetime values for this distance were taken from the ensemble experiments shown in Figure 1. We assumed that ssDNA‐ATTO542 is separated from the MXene surface by a cation layer adding an estimated height of 1 nm, according to prior molecular dynamic simulations.^[^ ^28^ ^]^ The τ_0_ values were extracted from the dye‐ssDNA adsorbed on glass. For the τ_0_ values of the DNA origami structures, we observed slight variations between the different nanostructures due to the varying neighboring nucleobases adjacent to the dye molecule^[^ ^50^ ^]^ in each NRO design. To account for these inter‐origami variations, we used the τ_0_ value of each individual NRO design to calculate the ET efficiency at different distances from the MXene surface.

The distance dependence of ET efficiency was fitted with $y=\frac{1}{(1+{\left(\frac{x}{d_{0}}\right)}^{a})\;}$, where *d*_0_ is the characteristic energy transfer distance (ET = 50%) and *a* is the distance dependent scaling power. The fit resulted in a *d*_0_ value of ≈2.71 nm (± 0.16) and a distance dependency of *d* ^−3^ (*a* = 2.84 ± 0.18). The latter can be understood with the following geometrical considerations: A *d* ^−6^ dependency is ascribed to point dipole‐to‐dipole coupling (FRET), a *d* ^−4^ dependency is indicative of a point dipole‐to‐plane coupling (e.g., graphene energy transfer), and a *d* ^−3^ dependency occurs in the case of a point dipole‐to‐cube coupling. The assumption of quartic distance‐scaling law (due to MXenes being 2D materials) leads to a fit that does not follow the experimental trend, as shown in dashed lines in Figure 4B, while assuming a cubic scaling law allows achieving a proper fit of the experimental data, as depicted in dotted lines (Figure 4B). The cubic dependency reveals that the nonradiative energy transfer from an emitter to the MXene layered substrate is a bulk process, agreeing with prior works on transparent conductors and metals at short distances.^[^ ^12^, ^51^ ^]^

We previously observed that the thickness‐dependent quenching efficiency of MXenes (Figure 1) is notably less pronounced than in materials like graphene, where a second layer doubles the energy transfer rate.^[^ ^43^ ^]^ This is because of the short quenching dynamic range of MXenes, where the *d*_0_ value is 2.7 nm. Considering a *d*‐spacing value of 1.62 nm, our theoretical calculation shows a maximum difference in energy transfer efficiency of only 6.2% from one to two MXene layers (see “*Calculations on the effect of MXene multilayers on the energy transfer efficiency of ATTO 542”* in SI's Materials and Methods). Such relative difference is obtained when the dye is positioned at 3.4 nm from the surface, assuming that the quenching effect is additive between the layers (Figure S9, Supporting Information).

### 2.4 Single‐Molecule Sensing of Dye‐Labeled Supported Lipid Bilayers with MXene ET

**Figure 5:**
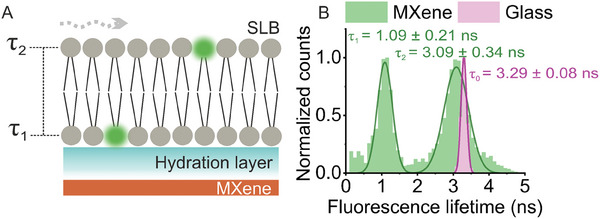
Single‐molecule leaflet‐resolved sensing of dye‐labeled supported lipid bilayers (SLBs) with MXene ET: A) Simplified scheme of SLBs composed of 1,2‐dioleoyl‐sn‐glycero‐3‐phosphocholine (DOPC) and 1,2‐dioleoyl‐sn‐glycero‐3‐phosphoethanolamine (DOPE) (the latter labeled in the head group with ATTO 532) at a ratio of 99.95:0.05, respectively. The SLBs fused on MXene‐on‐glass substrates depict two states given by leaflet‐specific fluorescence lifetime values τ_1_ and τ_2_. Wavy dashed arrow indicates SLBs’ fluidity. B) Lifetime distributions of SLBs on MXene flakes (green bins, *n* = 3803 data points derived from single‐molecule bursts) and on glass alone (pink bins, *n* = 4106 data points derived from FLIM image analysis). Lifetime values were calculated via reconvolution fitting with measured IRF. The distributions were fitted using a Gaussian model. The average lifetime value and standard deviation of the distributions are displayed.

Ti_3_C_2_T*_x_* MXene offers specific advantages as a quenching substrate: hydrophilicity, a nearly layer‐independent ET response, and sensitivity within short distances. Such properties can be effectively utilized to sense dyes within lipid bilayers, which are mobile assemblies of only ≈5 nm of thickness. We assembled dye‐labeled supported lipid bilayers (SLBs) composed of 1,2‐dioleoyl‐sn‐glycero‐3‐phosphocholine (DOPC) and ATTO 532‐labeled 1,2‐dioleoyl‐sn‐glycero‐3‐phosphoethanolamine (DOPE) (99.95:0.05) onto films of MXene flakes via vesicle fusion. We aimed to resolve two distinct lifetime states in the same sample: one corresponding to the dyes located in the head groups of the lower leaflet (τ_1_) and another to those found in the upper leaflet (τ_2_) (**Figure** **5**A). These states were predicted to be far apart in terms of ET efficiency. The *z*‐distances of dye‐labeled lipids were evaluated using the distance dependence of MXene ET, given by $z=d_{0}\;{\left(\frac{1}{1\;-\;\frac{\tau }{\tau_{0}}\;}-1\right)}^{\frac{1}{3}}$, where *d*_0_ = 2.7 nm and τ_0_ = 3.29 ns.

Due to lipid mobility, single‐molecule bursts of labeled lipids passing through the focal volume were recorded on MXene flakes, identified via FLIM (Figure S10, Supporting Information). We chose an optimal ratio of labeled to unlabeled lipids to ensure a predominance of unlabeled lipids, enabling the measurement of single‐molecule bursts. We then calculated the fluorescence lifetime per 50 ms binning time, using a cutoff threshold of 400 photons (8 kHz) to minimize background interference. Figure 5B shows the fitted distribution of the resulting lifetime values, rendering the following mean values of τ_1_ and τ_2_ (± error of the fit): 1.09 ± 0.01 ns and 3.09 ± 0.02 ns. These values were converted to *z* distances *z*_1_ and *z*_2_ (2.14 ± 0.13 nm and 6.62 ± 0.43 nm, respectively) using the equation above and the bilayer thickness (Δ*z*) was found to be 4.48 ± 0.33 nm. Despite our method's limitations, such as unaccounted effects from the lipid environment on dye orientation and refractive index, and predominantly short lifetime populations found on some MXene areas, our determined thickness value matches well with established methods. These include: 1) A quantitative differential interference contrast microscopy study, which recorded a 4.52 nm thickness for a bilayer (unaffected by substrate effects) using multilamellar dye‐labeled lipid films on glass;^[^ ^52^ ^]^ 2) high‐resolution x‐ray techniques, which found 4.53 nm for fully hydrated multilamellar^[^ ^53^ ^]^ and 4.48 nm for unilamellar stacked layers (the latter measured at 30°C);^[^ ^54^ ^]^ and 3) force spectroscopy, which measured 4.60 nm for SLBs on mica.^[^ ^55^ ^]^ Additionally, the hydration layer thickness (*z*_1_) separating SLBs from the MXene substrate is consistent with values for silica surfaces.^[^ ^4^ ^]^ This agreement, achieved at the single‐molecule level, is particularly encouraging and, to our knowledge, a novel achievement.

## 3 Conclusion

We presented the first study on MXene‐induced nonradiative energy transfer in the green spectral range. Using glycine‐modified DNA oligonucleotides, we immobilized DNA origami structures on MXene substrates, placing freely rotating fluorescent molecules at controlled heights. Our single‐molecule fluorescence lifetime results showed a *d* ^−3^ dependence of the energy transfer from a dye to MXene, with a 50% energy transfer distance (*d*_0_) of 2.7 ± 0.2 nm. This *d*_0_ is six times smaller than that of graphene, which is beneficial for the study of supported lipid bilayers given MXenes’ hydrophilicity. We were able to retrieve bilayer thickness values consistent with established methods using MXene energy transfer. Our study advances the integration of 2D materials with single‐molecule fluorescence methods, which hold significant potential for various applications, including biophysical sensing.

## Conflict of Interest

The authors declare no conflict of interest.

## Supporting information



Supporting Information

## Data Availability

The data that support the findings of this study are available from the corresponding author upon reasonable request.

## References

[1] K. H. Drexhage , J. Lumin. 1970, 1–2, 693.

[2] R. R. Chance , A. H. Miller , A. Prock , R. Silbey , J. Chem. Phys. 1975, 63, 1589.

[3] T. Förster , Zeitschrift für Naturforschung B 1947, 2, 174.

[4] T. Chen , A. Ghosh , J. Enderlein , Nano Lett. 2023, 23, 2421. 36706024 10.1021/acs.nanolett.2c04635 PMC10037415

[5] G. Agam , C. Gebhardt , M. Popara , R. Mächtel , J. Folz , B. Ambrose , N. Chamachi , S. Y. Chung , T. D. Craggs , M. de Boer , D. Grohmann , T. Ha , A. Hartmann , J. Hendrix , V. Hirschfeld , C. G. Hübner , T. Hugel , D. Kammerer , H.‐S. Kang , A. N. Kapanidis , G. Krainer , K. Kramm , E. A. Lemke , E. Lerner , E. Margeat , K. Martens , J. Michaelis , J. Mitra , G. G. Moya Muñoz , R. B. Quast , et al., Nat. Methods 2023, 20, 523. 36973549 10.1038/s41592-023-01807-0 PMC10089922

[6] B. Hellenkamp , S. Schmid , O. Doroshenko , O. Opanasyuk , R. Kühnemuth , S. Rezaei Adariani , B. Ambrose , M. Aznauryan , A. Barth , V. Birkedal , M. E. Bowen , H. Chen , T. Cordes , T. Eilert , C. Fijen , C. Gebhardt , M. Götz , G. Gouridis , E. Gratton , T. Ha , P. Hao , C. A. Hanke , A. Hartmann , J. Hendrix , L. L. Hildebrandt , V. Hirschfeld , J. Hohlbein , B. Hua , C. G. Hübner , E. Kallis , et al., Nat. Methods 2018, 15, 669. 30171252 10.1038/s41592-018-0085-0 PMC6121742

[7] W. R. Algar , N. Hildebrandt , S. S. Vogel , I. L. Medintz , Nat. Methods 2019, 16, 815. 31471616 10.1038/s41592-019-0530-8

[8] H. S. Chung , J. M. Louis , W. A. Eaton , Biophys. J. 2010, 98, 696. 20159166 10.1016/j.bpj.2009.12.4322 PMC2820649

[9] N. Karedla , A. I. Chizhik , I. Gregor , A. M. Chizhik , O. Schulz , J. Enderlein , ChemPhysChem 2014, 15, 705. 24478241 10.1002/cphc.201300760

[10] A. I. Chizhik , J. Rother , I. Gregor , A. Janshoff , J. Enderlein , Nat. Photon 2014, 8, 124.

[11] A. Ghosh , A. I. Chizhik , N. Karedla , J. Enderlein , Nat. Protoc. 2021, 16, 3695. 34099942 10.1038/s41596-021-00558-6

[12] R. J. Moerland , J. P. Hoogenboom , Optica 2016, 3, 112.

[13] L. Hauke , S. Isbaner , A. Ghosh , I. Guido , L. Turco , A. I. Chizhik , I. Gregor , N. Karedla , F. Rehfeldt , J. Enderlein , ACS Nano 2023, 17, 8242. 36995274 10.1021/acsnano.2c12372 PMC10173696

[14] N. Füllbrunn , Z. Li , L. Jorde , C. P. Richter , R. Kurre , L. Langemeyer , C. Yu , C. Meyer , J. Enderlein , C. Ungermann , J. Piehler , C. You , eLife 2021, 10, e62501. 33513092 10.7554/eLife.62501 PMC7847308

[15] I. Kamińska , J. Bohlen , R. Yaadav , P. Schüler , M. Raab , T. Schröder , J. Zähringer , K. Zielonka , S. Krause , P. Tinnefeld , Adv. Mater. 2021, 33, 2101099. 33938054 10.1002/adma.202101099 PMC11468539

[16] A. M. Szalai , G. Ferrari , L. Richter , J. Hartmann , M.‐Z. Kesici , B. Ji , K. Coshic , A. Jaeger , A. Aksimentiev , I. Tessmer , I. Kamińska , A. M. Vera , P. Tinnefeld , bioRxiv 2023, 11, 567962. 10.1038/s41592-024-02498-x 39516563

[17] T. Chen , N. Karedla , J. Enderlein , Nat. Commun. 2024, 15, 1789. 38413608 10.1038/s41467-024-45822-x PMC10899616

[18] A. Ghosh , A. Sharma , A. I. Chizhik , S. Isbaner , D. Ruhlandt , R. Tsukanov , I. Gregor , N. Karedla , J. Enderlein , Nat. Photonics 2019, 13, 860.

[19] M. Naguib , M. Kurtoglu , V. Presser , J. Lu , J. Niu , M. Heon , L. Hultman , Y. Gogotsi , M. W. Barsoum , Adv. Mater. 2011, 23, 4248. 21861270 10.1002/adma.201102306

[20] B. Anasori , M. R. Lukatskaya , Y. Gogotsi , Nat. Rev. Mater. 2017, 2, 16098.

[21] B. Xu , M. Zhu , W. Zhang , X. Zhen , Z. Pei , Q. Xue , C. Zhi , P. Shi , Adv. Mater. 2016, 28, 3333. 26924616 10.1002/adma.201504657

[22] F. Shahzad , M. Alhabeb , C. B. Hatter , B. Anasori , S. Man Hong , C. M. Koo , Y. Gogotsi , Science 2016, 353, 1137. 27609888 10.1126/science.aag2421

[23] Z. Liu , H. N. Alshareef , Adv. Electron. Mater. 2021, 7, 2100295.

[24] S. Panuganti , L. V. Besteiro , E. S. Vasileiadou , J. M. Hoffman , A. O. Govorov , S. K. Gray , M. G. Kanatzidis , R. D. Schaller , J. Am. Chem. Soc. 2021, 143, 4244. 33688726 10.1021/jacs.0c12441

[25] L. Richter , A. M. Szalai , C. L. Manzanares‐Palenzuela , I. Kamińska , P. Tinnefeld , Adv. Mater. 2023, 35, 2303152. 10.1002/adma.202303152 37670535

[26] J. Zähringer , F. Cole , J. Bohlen , F. Steiner , I. Kamińska , P. Tinnefeld , Light Sci. Appl. 2023, 12, 70. 36898993 10.1038/s41377-023-01111-8 PMC10006205

[27] Q. Zhang , F. Wang , H. Zhang , Y. Zhang , M. Liu , Y. Liu , Anal. Chem. 2018, 90, 12737. 30350604 10.1021/acs.analchem.8b03083

[28] C. L. Manzanares‐Palenzuela , A. M. Pourrahimi , J. Gonzalez‐Julian , Z. Sofer , M. Pykal , M. Otyepka , M. Pumera , Chem. Sci. 2019, 10, 10010. 32055358 10.1039/c9sc03049b PMC6979399

[29] Z. Huang , B. Liu , J. Liu , Langmuir 2019, 35, 9858. 31265783 10.1021/acs.langmuir.9b01810

[30] S. Isbaner , N. Karedla , I. Kaminska , D. Ruhlandt , M. Raab , J. Bohlen , A. Chizhik , I. Gregor , P. Tinnefeld , J. Enderlein , R. Tsukanov , Nano Lett. 2018, 18, 2616. 29562123 10.1021/acs.nanolett.8b00425

[31] G. P. Acuna , M. Bucher , I. H. Stein , C. Steinhauer , A. Kuzyk , P. Holzmeister , R. Schreiber , A. Moroz , F. D. Stefani , T. Liedl , F. C. Simmel , P. Tinnefeld , ACS Nano 2012, 6, 3189. 22439823 10.1021/nn2050483

[32] C. Steinhauer , R. Jungmann , T. L. Sobey , F. C. Simmel , P. Tinnefeld , Angew. Chem., Int. Ed. 2009, 48, 8870. 10.1002/anie.200903308 19830751

[33] I. H. Stein , V. Schüller , P. Böhm , P. Tinnefeld , T. Liedl , ChemPhysChem 2011, 12, 689. 21308944 10.1002/cphc.201000781

[34] I. Kaminska , J. Bohlen , S. Rocchetti , F. Selbach , G. P. Acuna , P. Tinnefeld , Nano Lett. 2019, 19, 4257. 31251640 10.1021/acs.nanolett.9b00172

[35] C. Chen , M. Boota , P. Urbankowski , B. Anasori , L. Miao , J. Jiang , Y. Gogotsi , J. Mater. Chem. A 2018, 6, 4617.

[36] C. (John) Zhang , B. Anasori , A. Seral‐Ascaso , S.‐H. Park , N. McEvoy , A. Shmeliov , G. S. Duesberg , J. N. Coleman , Y. Gogotsi , V. Nicolosi , Adv. Mater. 2017, 29, 1702678. 10.1002/adma.201702678 28741695

[37] M. Shekhirev , C. E. Shuck , A. Sarycheva , Y. Gogotsi , Prog. Mater. Sci. 2021, 120, 100757.

[38] K. Prenger , Y. Sun , K. Ganeshan , A. Al‐Temimy , K. Liang , C. Dun , J. J. Urban , J. Xiao , T. Petit , A. C. T. van Duin , D. Jiang , M. Naguib , ACS Appl. Energy Mater. 2022, 5, 9373.

[39] Q. Gao , W. Sun , P. Ilani‐Kashkouli , A. Tselev , P. R. C. Kent , N. Kabengi , M. Naguib , M. Alhabeb , W.‐Y. Tsai , A. P. Baddorf , J. Huang , S. Jesse , Y. Gogotsi , N. Balke , Energy Environ. Sci. 2020, 13, 2549.

[40] J. Kim , L. J. Cote , F. Kim , J. Huang , J. Am. Chem. Soc. 2010, 132, 260. 19961229 10.1021/ja906730d

[41] D. A. Panova , G. I. Tselikov , G. A. Ermolaev , A. V. Syuy , D. S. Zimbovskii , O. O. Kapitanova , D. I. Yakubovsky , A. B. Mazitov , I. A. Kruglov , A. A. Vyshnevyy , A. V. Arsenin , V. S. Volkov , Opt. Lett. 2024, 49, 25. 38134143 10.1364/OL.503636

[42] J. K. El‐Demellawi , S. Lopatin , J. Yin , O. F. Mohammed , H. N. Alshareef , ACS Nano 2018, 12, 8485. 30020767 10.1021/acsnano.8b04029

[43] A. Raja , A. Montoya−Castillo , J. Zultak , X.‐X. Zhang , Z. Ye , C. Roquelet , D. A. Chenet , A. M. van der Zande , P. Huang , S. Jockusch , J. Hone , D. R. Reichman , L. E. Brus , T. F. Heinz , Nano Lett. 2016, 16, 2328. 26928675 10.1021/acs.nanolett.5b05012

[44] Z. Chen , S. Berciaud , C. Nuckolls , T. F. Heinz , L. E. Brus , ACS Nano 2010, 4, 2964. 20402475 10.1021/nn1005107

[45] H. Kim , M. I. Nugraha , X. Guan , Z. Wang , M. K. Hota , X. Xu , T. Wu , D. Baran , T. D. Anthopoulos , H. N. Alshareef , ACS Nano 2021, 15, 5221. 33635642 10.1021/acsnano.0c10471

[46] V. Mauchamp , M. Bugnet , E. P. Bellido , G. A. Botton , P. Moreau , D. Magne , M. Naguib , T. Cabioc'h , M. W. Barsoum , Phys. Rev. B 2014, 89, 235428.

[47] J. J. Schmied , M. Raab , C. Forthmann , E. Pibiri , B. Wünsch , T. Dammeyer , P. Tinnefeld , Nat. Protoc. 2014, 9, 1367. 24833175 10.1038/nprot.2014.079

[48] N. V. Voigt , T. Tørring , A. Rotaru , M. F. Jacobsen , J. B. Ravnsbæk , R. Subramani , W. Mamdouh , J. Kjems , A. Mokhir , F. Besenbacher , K. V. Gothelf , Nat. Nanotech. 2010, 5, 200. 10.1038/nnano.2010.5 20190747

[49] J. D. Gouveia , G. Novell‐Leruth , P. M. L. S. Reis , F. Viñes , F. Illas , J. R. B. Gomes , ACS Appl. Bio Mater. 2020, 3, 5913. 10.1021/acsabm.0c00621 35021819

[50] C. A. M. Seidel , A. Schulz , M. H. M. Sauer , J. Phys. Chem. 1996, 100, 5541.

[51] W. L. Barnes , J. Mod. Opt. 1998, 45, 661.

[52] D. Regan , J. Williams , P. Borri , W. Langbein , Langmuir 2019, 35, 13805. 31483674 10.1021/acs.langmuir.9b02538 PMC7007255

[53] S. Tristram‐Nagle , H. I. Petrache , J. F. Nagle , Biophys. J. 1998, 75, 917. 9675192 10.1016/S0006-3495(98)77580-0 PMC1299765

[54] J. Pan , S. Tristram‐Nagle , N. Kučerka , J. F. Nagle , Biophys. J. 2008, 94, 117. 17827241 10.1529/biophysj.107.115691 PMC2134881

[55] S. J. Attwood , Y. Choi , Z. Leonenko , Int. J. Mol. Sci. 2013, 14, 3514. 23389046 10.3390/ijms14023514 PMC3588056

