# Supplementary material for: 2D Titanium Carbide MXene and Single‐Molecule Fluorescence: Distance‐Dependent Nonradiative Energy Transfer and Leaflet‐Resolved Dye Sensing in Lipid Bilayers
Source: Adv Mater. 2024 Oct 24;36(49):2411724. doi: 10.1002/adma.202411724 (PMC11619223; doi:10.1002/adma.202411724)
Supplement: Supplementary file 1 — Supporting Information [file ADMA-36-2411724-s001.pdf]

ADVANCED MATERIALS

Supporting Information

for *Adv. Mater.*, DOI 10.1002/adma.202411724

2D Titanium Carbide MXene and Single-Molecule Fluorescence: Distance-Dependent Nonradiative Energy Transfer and Leaflet-Resolved Dye Sensing in Lipid Bilayers

Lorena Manzanares, Dahnan Spurling, Alan M. Szalai, Tim Schröder, Ece Büber, Giovanni Ferrari, Martin R. J. Dagleish, Valeria Nicolosi and Philip Tinnefeld**

2D titanium carbide MXene and single-molecule fluorescence: Distance-dependent nonradiative energy transfer and leaflet-resolved dye sensing in lipid bilayers

Lorena Manzanares[a,b], Dahnan Spurling[d], Alan M. Szalai[b,c], Tim Schröder[b], Ece Büber[b], Giovanni Ferrari[b], Martin Dagleish[b], Valeria Nicolosi[d], Philip Tinnefeld[b]**

L. Manzanares
[a]Univ. Lille, CNRS, Centrale Lille, Univ. Polytechnique Hauts-de-France, UMR 8520 - IEMN - Institut d'Electronique de Microélectronique et de Nanotechnologie, F-59000, Lille, France
E-mail: cmanzana@centralelille.fr

L. Manzanares, A. M. Szalai, T. Schröder, E. Büber, G. Ferrari, P. Tinnefeld
[b]Department of Chemistry and Center for NanoScience, Ludwig-Maximilians-University, Butenandtstraße 5-13, 81377, Munich, Germany
E-mail: cmanzana@centralelille.fr, philip.tinnefeld@cup.lmu.de

A. M. Szalai
[c]CONICET Centro de Investigaciones en Bionanociencias (CIBION), Polo Científico Tecnológico Godoy Cruz 2390, Buenos Aires, Argentina

D. Spurling, V. Nicolosi
[d]School of Chemistry, Trinity College Dublin, Dublin 2, Ireland

Keywords: MXene, DNA origami, supported lipid bilayers, energy transfer, single-molecule, fluorescence microscopy, 2D materials

Materials and methods

Synthesis of $Ti_3C_2T_x$ flakes. The synthesis of MXene flakes was carried out following prior protocols,[1] with slight modifications. We used MAX phase Ti_3AlC_2 with excess aluminium, which is known to result in low defect concentrations in the final MXene.[2] Briefly, a 9M HCl solution (Sigma, 20 ml) was mixed with 1.6 g of LiF powder (Sigma) in a PTFE vented container set in an oil bath. For LiF dissolution, stirring was set at 400 rpm for 10 minutes. After that, 1 g of the MAX phase Ti_3AlC_2 (Carbon-Ukraine Ltd.) was slowly introduced to the mix, to avoid excessive heating. The solution was then stirred for 24 h at 35°C at 400 rpm for complete MAX phase etching. Next, the solution was diluted with deionized water to a volume of 40 ml and centrifuged for 5 mat 2800 *g* (5000 rpm). The supernatant was discarded, and the remaining sediment was dispersed again in 40 ml deionized water, and the process was repeated until a pH > 6 was achieved. To obtain mono- and few-layer $Ti_3C_2T_x$ flakes, the solution was vortexed for 30 m, then centrifuged for 30 mat 250 g (1500 rpm). The supernatant, now with monolayer flakes, was separated. The last step involved centrifugation for an hour at 2800 g (5000 rpm) to concentrate the monolayer flakes in the sediment, which was then redispersed to produce a stock solution of $Ti_3C_2T_x$ flakes. Vacuum filtration of the stock $Ti_3C_2T_x$ dispersions was used to create freestanding films from which the concentration could be determined. We then carried out EDX, Raman, and XRD analysis on these films.

Fabrication of thin films. Glass cover slips of thickness 1.5 H were first cleaned using ultrasonication in water followed by isopropanol. The slips were then treated in a bath containing 3M NaOH for 15 minutes to remove organic contaminants and increase the hydrophilicity of the glass. The treated slips were rinsed three times with distilled water and dried using compressed air. To coat, the as-prepared $Ti_3C_2T_x$ dispersions were diluted to a concentration of 2 mg mL^{-1} and vortex mixed for 3 minutes to homogenize. A benchtop spin coater (Ossila) was used to produce the thin films, rotating at 800 rpm for 10 s followed by 2500 rpm for 60 s. The coated slides were then placed in a vacuum oven at 90°C for 1 h to remove excess water from the thin films.

Synthesis of DNA origami nanopositioners. The rectangular DNA origami template was designed using caDNAno software, with details available in Fig. S4. A 7249-nucleotide long scaffold (Table S5) was extracted from M13mp18 bacteriophages. Staple strands were acquired from Eurofins Genomics GmbH. ATTO 542 modified oligos were bought from biomers.net. For the folding process, scaffold (20 nM) and oligos (including both internal and external ATTO 542 labeled oligos) listed in Table S2-S4 were combined at specified final concentrations:

unmodified staples were added in 10-fold excess to the scaffold's concentration and modified staples were added in 25- and 50-fold excess in the case of internal and external labeling strands, respectively. TAE-Mg^{2+}-20 (see Table S1 for the list of buffers) was used as the folding buffer. A thermal annealing cycle (1 °C min^{-1} ramp starting from 95 °C) was employed to facilitate folding. The DNA origami constructs were then isolated through gel electrophoresis using a 1.5% agarose gel in a TAE-Mg^{2+}-12.5 buffer, run at 60 V for 90 minutes in an ice-cooled gel box. The gel was stained with ROTI®GelStain (Carl Roth). DNA origami structures were visible under UV illumination. The desired band was excised, and the DNA origami constructs were mechanically retrieved from the gel. Samples were preserved at -20°C for future analysis. NanoDrop measurements were carried out to determine the concentration of purified DNA origami.

Immobilization of DNA origami nanopositioners on MXene films. DNA origami solutions were prepared in PBS-Na^+ at a final concentration of 200 pM and saturated with argon gas to avoid oxidation of MXenes[3] prior to surface incubation. MXene glass films were flushed with a stream of argon gas prior to use. A silicone-gasketed chamber was glued onto the films (SecureSeal™ Hybridization Chambers from Grace Bio-Labs) by placing the bottom of the glass slides on a hot plate at 100 °C for 1 min. 150 µL of the argon-filled DNA origami solution was added in the chamber and sealed to protect MXenes from oxidation. After 1 h of incubation at RT, the surface was washed 3 times with PBS-Na^+-Tween.

Immobilization of DNA origami nanopositioners on glass substrates. The glass surface was cleaned by UV-ozone treatment for 30 min, followed by a 1-min KOH 1 M incubation and rinsing with Milli-Q water. A silicone-gasketed chamber was thermally glued onto the clean glass as described in the prior paragraph. Next, biotin labeled bovine serum albumin (BSA) (Sigma-Aldrich Chemie GmbH) was added (1 mg mL^{-1}) to the chamber, incubated, washed 4 times with PBS 1x, followed by 1 mg mL^{-1} NeutrAvidin (Sigma-Aldrich Chemie GmbH) incubation and the same washing procedure as before. Biotin-labeled NROs were added to the BSA coated surface at a concentration of 50 pM in TAE-Mg^{2+} and washed and kept with PBS-Na^+-Tween to mimic the ionic conditions at which the MXene samples were measured. All the incubations stated here were 3 to 5 min-long.

Preparation of supported lipid bilayers (SLBs) on MXene-glass films and bare glass. Large unilamellar vesicles (LUVs) composed of DOPC (1,2-dioleoyl-sn-glycero-3-phosphocholine, purchased from Avanti Polar Lipids, USA) containing ATTO-532-DOPE (1,2-dioleoyl-sn-glycero-3-phosphoethanolamine, purchased from ATTO-TEC GmbH, Germany) in 99.95:0.05

percent ratio. All lipid stocks were dissolved in chloroform. The 99.95:0.05 mixture was subsequently dried under a nitrogen stream, followed by evaporation of the remaining chloroform under vacuum desiccation for ~3 hours. The lipid film was rehydrated in a LUV buffer containing 5 mM Tris, 1 mM EDTA, 0.5 mM Trolox and 150 mM NaCl at pH 7.0, resulting in a final lipid concentration of 2.5 mM. The solution underwent seven freeze-thaw cycles using liquid nitrogen and a 70°C water bath and then extruded through Nucleopore PC membranes with a 100-nm pore size (Whatman, Cytiva Ltd.) using a LiposoFast Basic extruder (Avestin, INC.). For the preparation of the SLBs on clean MXene films on glass and bare glass surfaces, 150 µL of 0.1 M $CaCl_2$ was added to surface-glued SecureSeal™ Hybridization Chambers (Grace Bio-Labs) for 2-3 min. The surfaces were cleaned prior to incubation with calcium as described in prior sections. The whole volume was then removed and 6 µL of the fresh calcium solution were re-added to the chamber, followed by 150 µL of 2 mM LUVs. The vesicles were incubated on the surface for 20 min at RT, followed by 4 washes with EDTA 1 mM and 10 subsequent washes with LUV buffer. The chambers were left with LUV buffer and equilibrated for 30 min for the SLBs to stabilize.

Single-molecule confocal fluorescence microscopy measurements. All tests were conducted using a custom confocal microscope designed around the Olympus IX71 inverted microscope framework. The DNA-origami constructs were illuminated using a pulsed laser (532 nm, LDH-P-FA-530B from PicoQuant GmbH). This laser was linked to a single-mode fiber (P3-488PM-FC, Thorlabs GmbH) to produce a Gaussian beam. We achieved circular polarization using a linear polarizer (LPVISE100-A, Thorlabs GmbH) followed by a quarter-wave plate (AQWP05M- 600, Thorlabs GmbH). The beam was then concentrated on the sample using an oil-immersion objective (UPLSAPO100XO, NA 1.40, Olympus Deutschland GmbH). The position of the sample was adjusted using a piezo stage (P-517.3CD, Physik Instrumente (PI) GmbH & Co. KG) and controller (E-727.3CDA, Physik Instrumente (PI) GmbH & Co. KG). To differentiate between the emitted light and the excitation light, a dichroic beamsplitter (zt532/640rpc, Chroma) was used. The light then passed through a 50 µm pinhole (Thorlabs GmbH). Post this, the emission was divided using another dichroic beamsplitter (640 LPXR, Chroma) and directed towards the green detection pathway (Brightline HC582/75, AHF; RazorEdge LP 532, Semrock). This emission was then captured on an avalanche photodiode (SPCM-AQRH-14-TR, Excelitas), and the data was processed by a TCSPC unit from PicoQuant (HydraHarp400, PicoQuant). The entire apparatus was managed using the SymPhoTime64 software from Picoquant GmbH.

For the surface studies, we used a 532 nm pulsed excitation with a 40 MHz repetition rate. An excitation power of 2 μW was set for all specimens containing DNA origami structures and 3 μW for SLB measurements. However, for the fluorophore layer consisting of ssDNA-dye, the laser power was lowered to 20 nW.

Fluorescence lifetime data analysis. The data obtained with the TCSPC module was analyzed using custom-made Python software (scripts available at https://github.com/alanszalai/smPyFLIM). The TCSPC decays were fit *via* reconvolution with the measured instrument response function (IRF) using either the least squares method or the maximum likelihood estimation (MLE) method. Least squares was used for fitting all the DNA/DNA origami data (with a minimum of 1000 photons in the total time interval before photobleaching), while MLE was used for fitting the SLBs' lifetimes, which had lower photon counts. In the latter case, single-molecule bursts were fitted at 50 ms binning times with an intensity threshold of 400 photons to minimize background interference.

AFM characterization of MXene films and DNA origami nanostructures. AFM imaging was carried out on a NanoWizard® 3 ultra-AFM (JPK Instruments AG). The MXene films were imaged in dry conditions. The DNA origami structures were adsorbed on a freshly cleaved mica surface by 5-min pre-treatment of the surface with $NiCl_2$ 10 mM. Next, the mica was washed three times with ultra-pure water and air-dried. The origami solutions (1 nM) were incubated for 5 minutes and measurements were performed with a BL-AC40TS-C2 cantilever from Olympus in PBS-Na^+ buffer.

Estimation of the glycine-linker end-to-end distance. In order to estimate the glycine linker end-to-end distance, we use the freely rotating chain model (FRC), which extends the freely jointed chain (FJC) model by the Flory's characteristics, which accounts for the "stiffness" of the linker. The equation is given as:

$$\langle R^2 \rangle = n \cdot l^2 \cdot \left(\frac{1 + \cos(\theta)}{1 - \cos(\theta)} \right)$$

With $\langle R^2 \rangle$ as the mean squared distance, the number of segments n, the length of one segment l and the torsion angle θ.

Given, that the terminal amine of the triglycine linkers binds to the MXene, the linker has 15 chain segments ($n = 15$). The length of the carbon-carbon bond is $l^2 = 153\, pm$. The bond angle in this chain is between 109.5° ($\theta = 70.5°$) for a carbon-carbon-carbon bond and 120°

($\theta = 60.0°$) for an amine angle. Therefore, we can calculate the root-mean-square distance to be $\sqrt{\langle R^2 \rangle}(\theta = 70.5°) = 0.82\ nm$ and $\sqrt{\langle R^2 \rangle}(\theta = 60°) = 0.98\ nm$.

Acquisition of the emission spectrum. The fluorescence spectrum was acquired on a Spectrofluorometer FS5 (Edinburgh Instruments). The dyes were excited with 532 nm central wavelength with a 2 nm slit width. The emission was recorded with a 1 nm step size and 5 seconds integration time from 540 to 700 nm with a 1 nm slit width. The sample holder was a high precision cell with a 1.5 × 1.5 mm light path from HellmaAnalytics. The cell was cleaned with a 1 % Hellmanex (HellmaAnalytics) solution before for 24 hours. Afterwards rinsed 10 times with MilliQ water and passivated with a 2.5 mg/mL BSA in PBS solution for 1 minute. Afterwards the cell was rinsed three additional times with PBS. The DNA origami solution ($NRO_{No\text{-}Gly}$ 0 bp) was placed in the cell afterwards. The temperature in the experiment was 20 °C.

Calculations on the effect of MXene multilayers on the energy transfer efficiency of ATTO 542. The energy transfer rate constant to the MXene can be calculated according to:

$$k_{ET}(d) = \frac{1}{\tau_0}\left(\frac{d_0}{d}\right)^3$$

Where $k_{ET}(d)$ is the distance dependent energy transfer efficiency, τ_0 is the donor's fluorescence lifetime without the MXene nearfield, d_0 is the characteristic energy transfer distance and d is the distance to the MXene surface.

The XRD-derived *d*-spacing value for MXene films in a Mg^{2+} buffer is 1.62 nm. Therefore, we assume the energy transfer efficiency to the second MXene layer to be:

$$k_{ET_2}(d) = \frac{1}{\tau_0}\left(\frac{d_0}{d + 1.62\ nm}\right)^3$$

Every additional layer will add additional 1.62 nm distance.

Therefore, we calculate the energy transfer efficiency for a single layer according to:

$$E = \frac{k_{ET}}{k_{ET} + \frac{1}{\tau_0}}$$

An additional layer will add an energy transfer rate:

$$E = \frac{k_{ET_1} + k_{ET_2}}{k_{ET} + k_{ET_2} + \frac{1}{\tau_0}}$$

The result is depicted in figure S9.

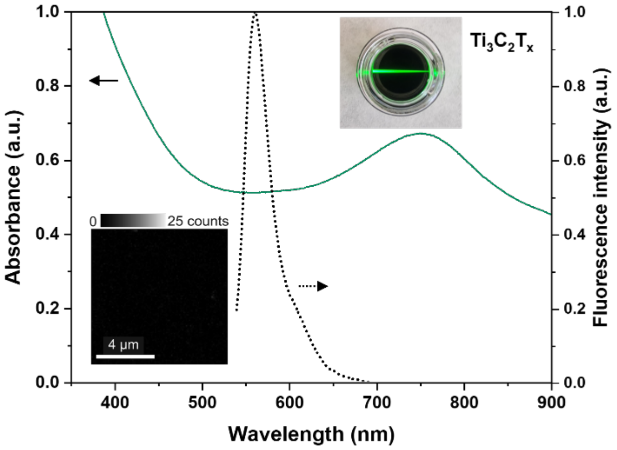

Figure S1. UV-Vis spectrum of a dispersion of $Ti_3C_2T_x$ flakes and fluorescence emission spectrum of ATTO-542-labeled NRO. Inlets: the Tyndall effect (top right) and autofluorescence of the MXene (bottom left) dispersion and films, respectively.

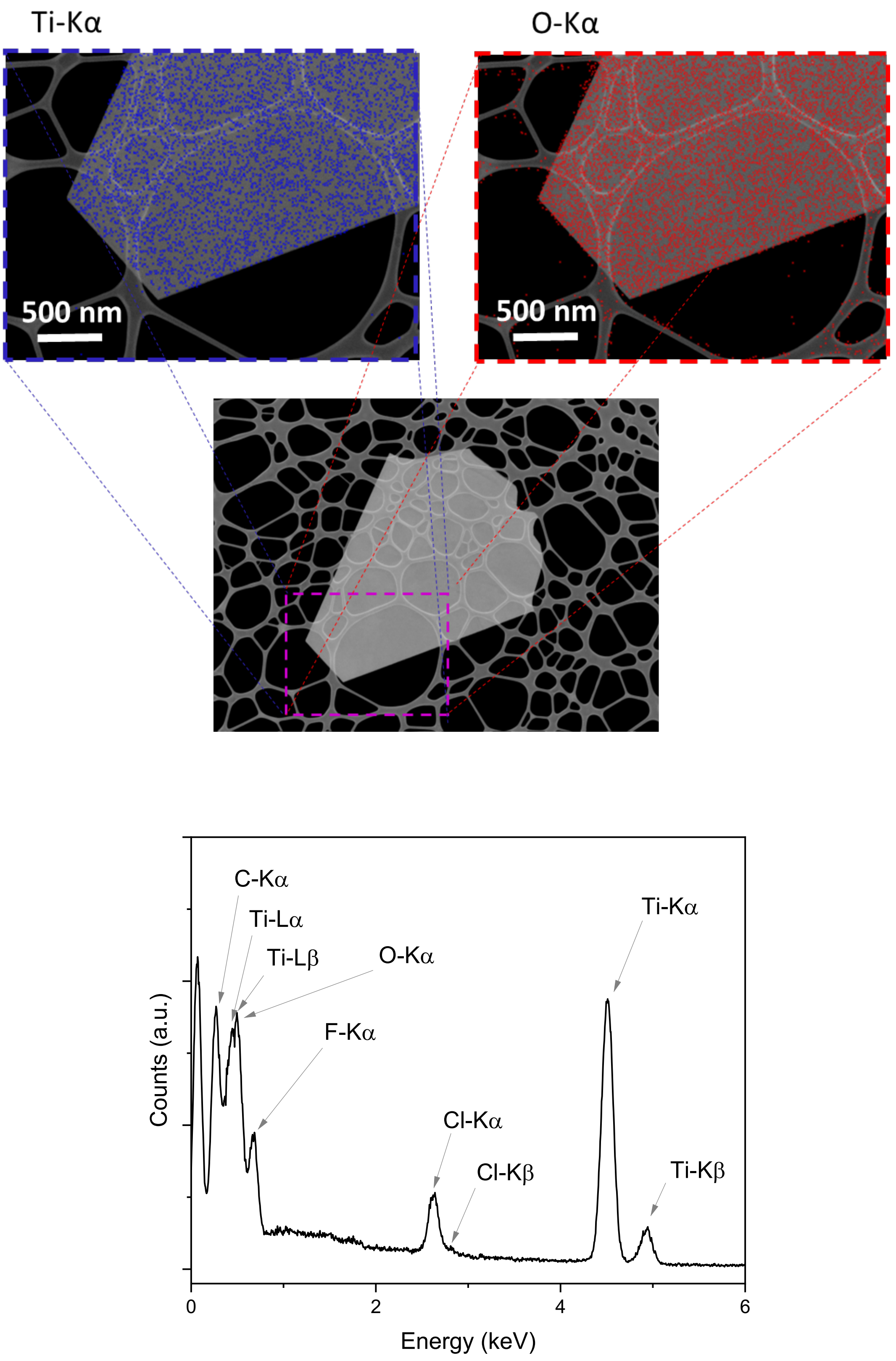

Figure S2. Representative SEM-EDX image (above) and elemental spectrum (below) of a single $Ti_3C_2T_x$ flake (10 kV acceleration voltage).

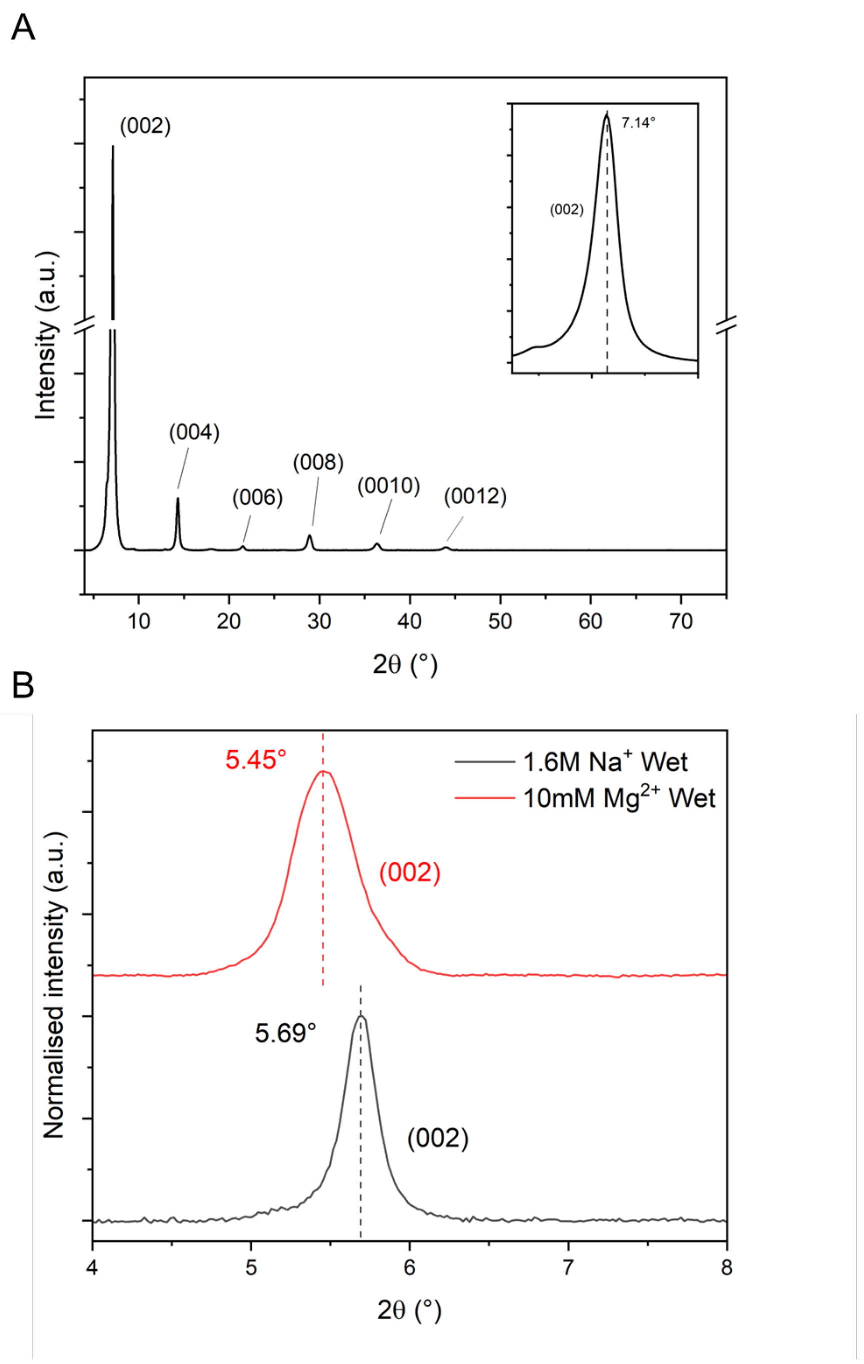

Figure S3. X-ray diffractograms of dry (A) and wet (B) spincast (44 mg mL^{-1}) films. The latter was soaked in a thin layer of electrolyte, mimicking the conditions in which the films were used for the energy transfer studies.

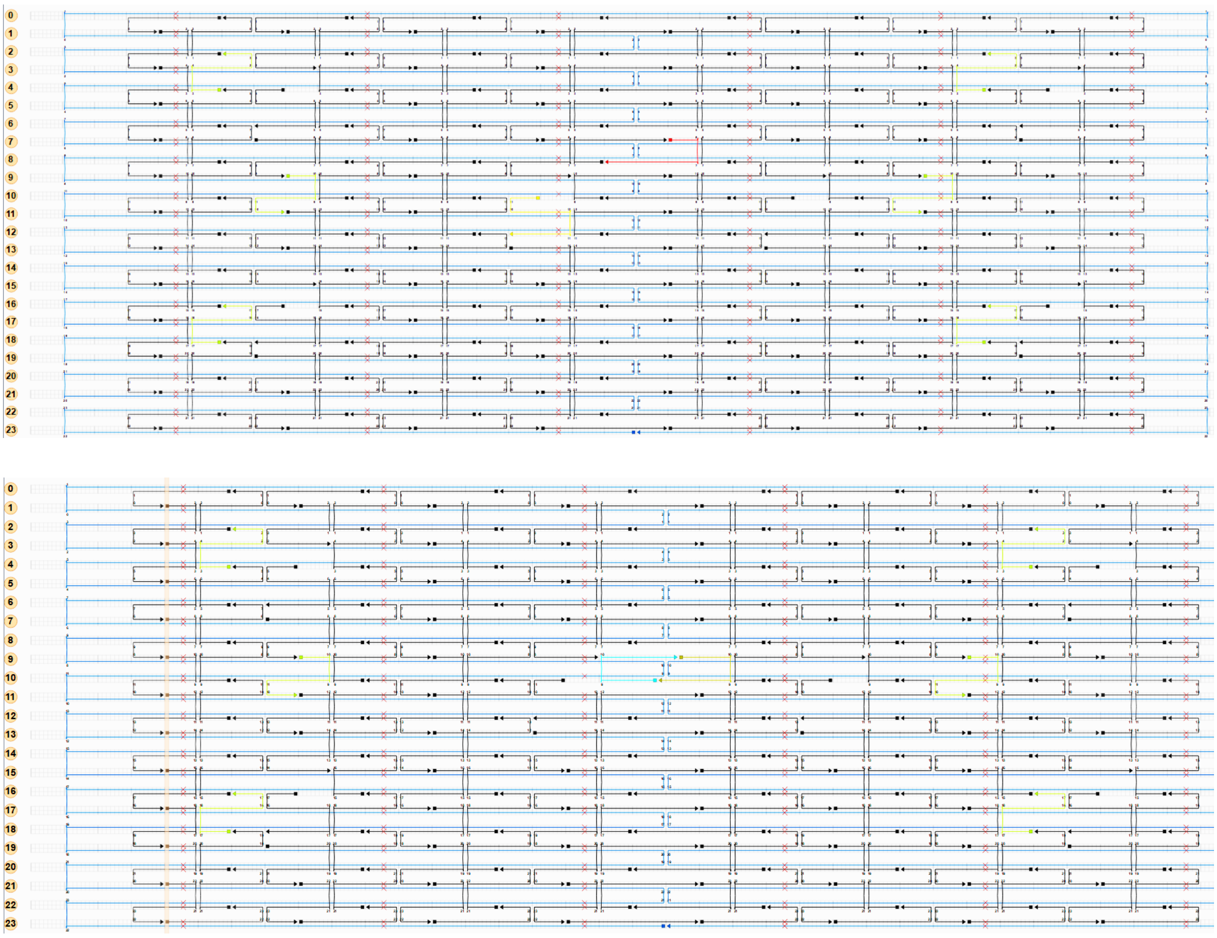

Figure S4. CaDNAno design of the NRO$_{\mathit{Gly}\text{-}3\mathrm{nm}}$ (above) and NRO protrusion sites for the design of 4 to 10 nm contour lengths (below). The long blue line represents the scaffold strand, and the short black lines represent unmodified staple strands. The square and the triangle ends represent 5’ and 3’ ends of the oligonucleotides. Green staples represent 5’-triglycine-oligonuleotides. The red staple is modified in the 5’ with up-facing ATTO 542 dyes. Light blue (5’) staples are the points of extensions or protrusions, modified with added sequences as shown in Table S4. Olive staples (3’) are extended with the complementary sequences of the light blue staples (called “unmodified hybridizing” in Table S4).

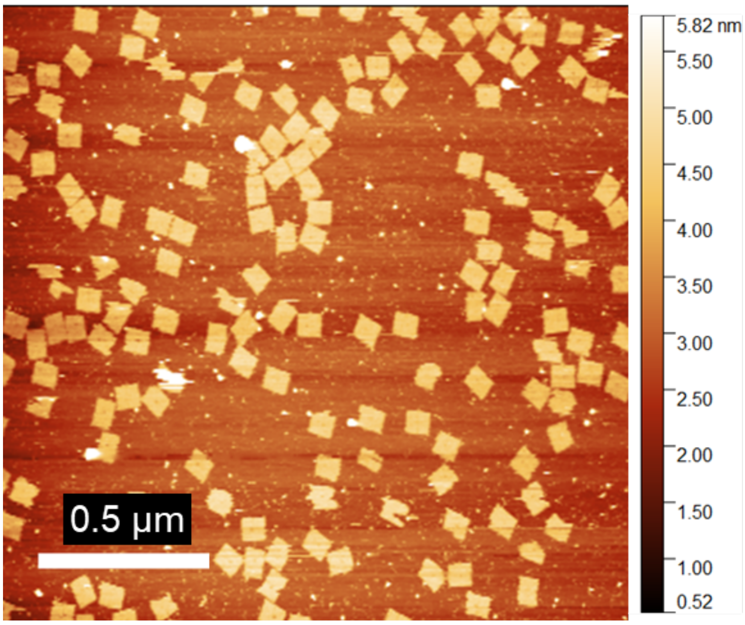

Figure S5. Representative atomic force micrographs of NROs in PBS-Na^+ buffer, adsorbed on a freshly-cleaved mica substrate.

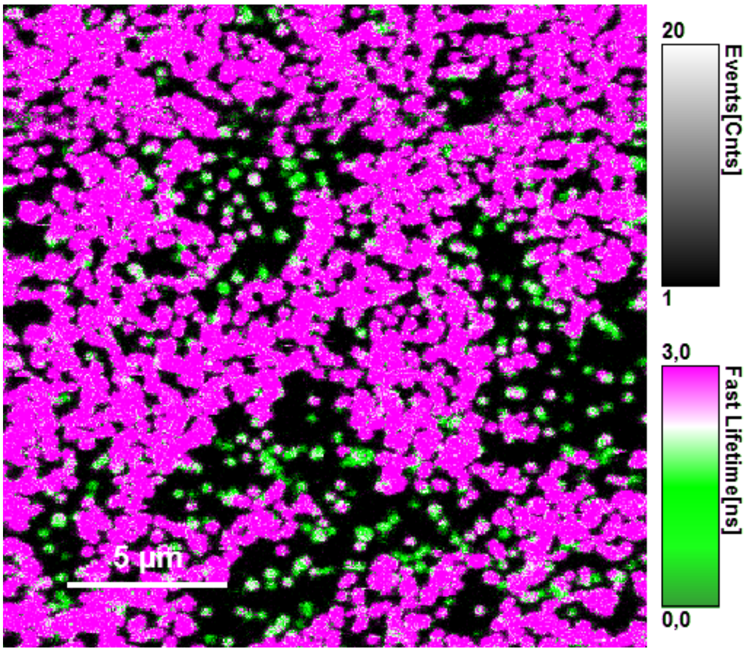

Figure S6. NRO immobilized on MXene films *via* Mg^{2+} bridges (in TAE-Mg^{2+}-12.5), without *Gly* linkers.

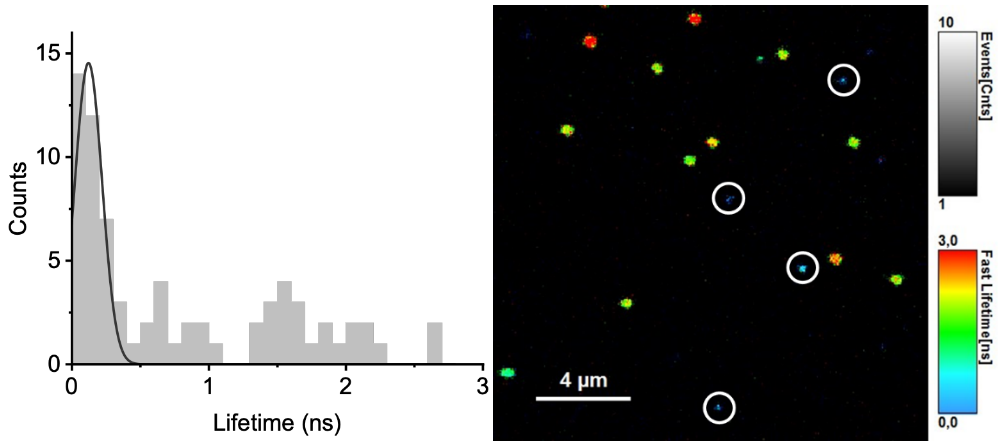

Figure S7. Height measurement of the 10 nm nanopositioner with a graphene substrate. The nanopositioner consisted of a 20-bp dsDNA strand protruding from NRO, where 6.8 nm is the contour length of the 20-bp dsDNA protrusion, 2 nm is the thickness of the NRO structure, and 1 nm is the substrate-NRO spacing. Left: Single molecule fluorescence lifetime distribution of ATTO 542-labeled NRO (10 nm) immobilized on monolayer graphene *via* single-stranded DNA extensions placed on the bottom of the structure. Right: FLIM images of color-coded lifetime of molecules on a graphene substrate. Encircled are NROs correctly oriented on graphene. Green and red spots are dyes located farther than expected from graphene possibly due to polymer residues, holes, or perpendicular orientations of NROs due to pi stacking interactions. The distribution (n = 72 molecules) was fitted with a Gaussian model, rendering an average lifetime of 0.12 ± 0.01 ns (R^2 = 0.61). Conversion to distances using d_0 = 17.7 nm and the d^4 power law[4] revealed a height of ~8 nm from the surface instead of 10 nm, suggesting that the double stranded protrusion stands with an average tilting angle of ~49 °.

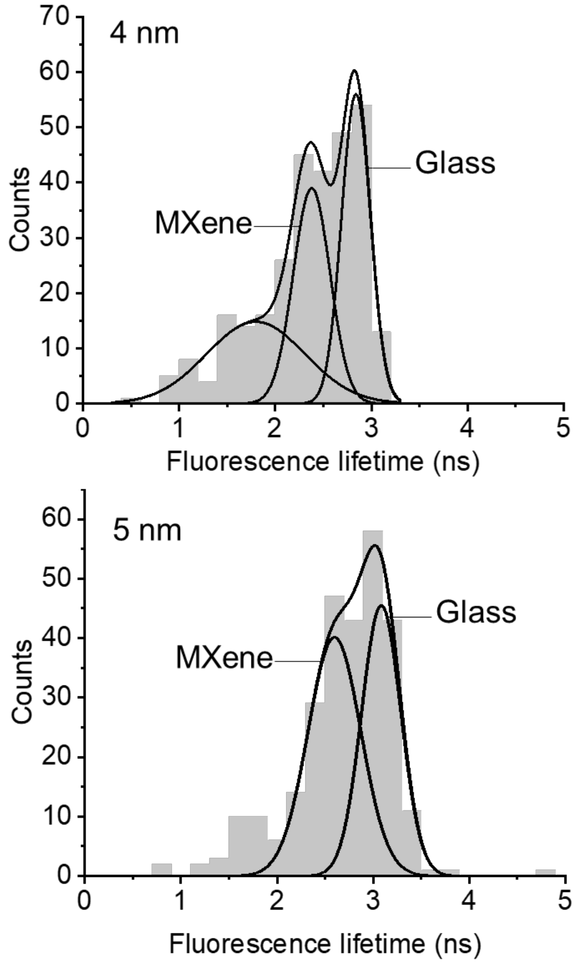

Figure S8. Single molecule fluorescence lifetime distribution of ATTO 542-labeled NRO (4 and 5 nm protrusions) immobilized on MXene flakes showing glass-contributing lifetimes. The distributions ($n \approx 280$ molecules) were fitted with a Gaussian model, rendering average lifetimes of 2.4 ± 0.2 ns and 2.8 ± 0.2 ns on MXene and glass areas for the 4 nm structure, respectively ($R^2 = 0.98$). For the 5 nm NRO, the average lifetimes were 2.6 ± 0.3 ns and 3.1 ± 0.2 ns on MXene and glass areas, respectively ($R^2 = 0.96$). The lifetime of ATTO 542 on glass areas inside MXene films were lower than those from BSA-biotin-NeutrAvidin glass surfaces used as reference measurements.

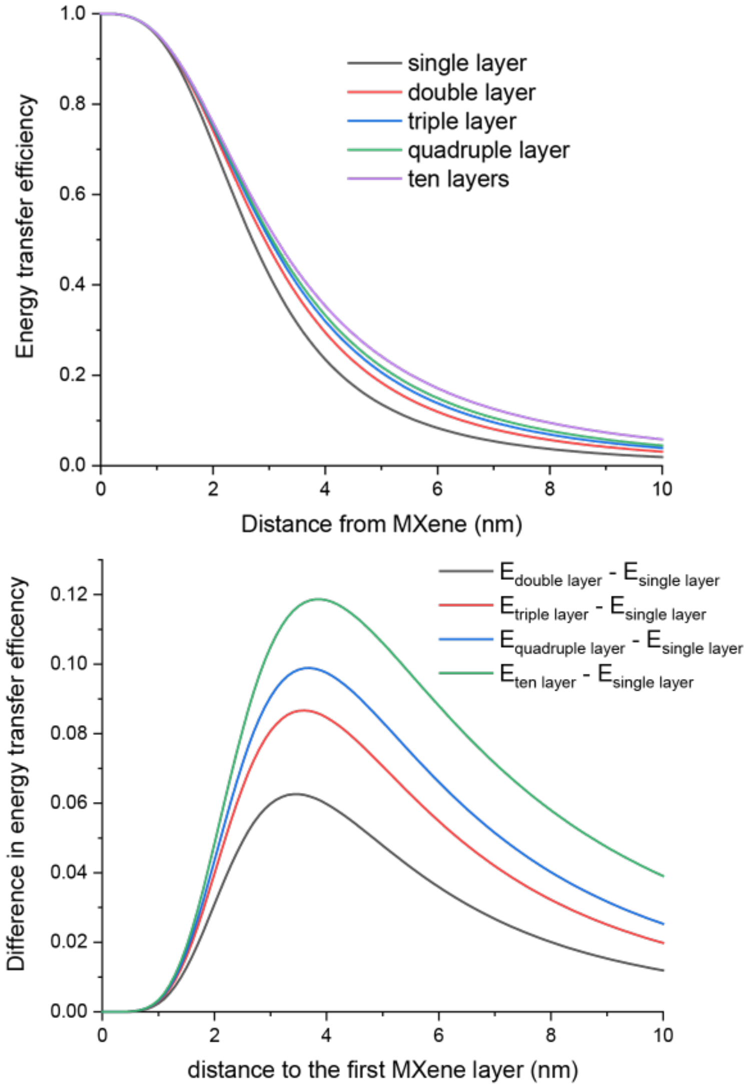

Figure S9. Effect of MXene multilayers on energy transfer efficiency with respect to the dye-MXene distance (above) and the differences of energy transfer efficiency values between layers as a function of the distance (below).

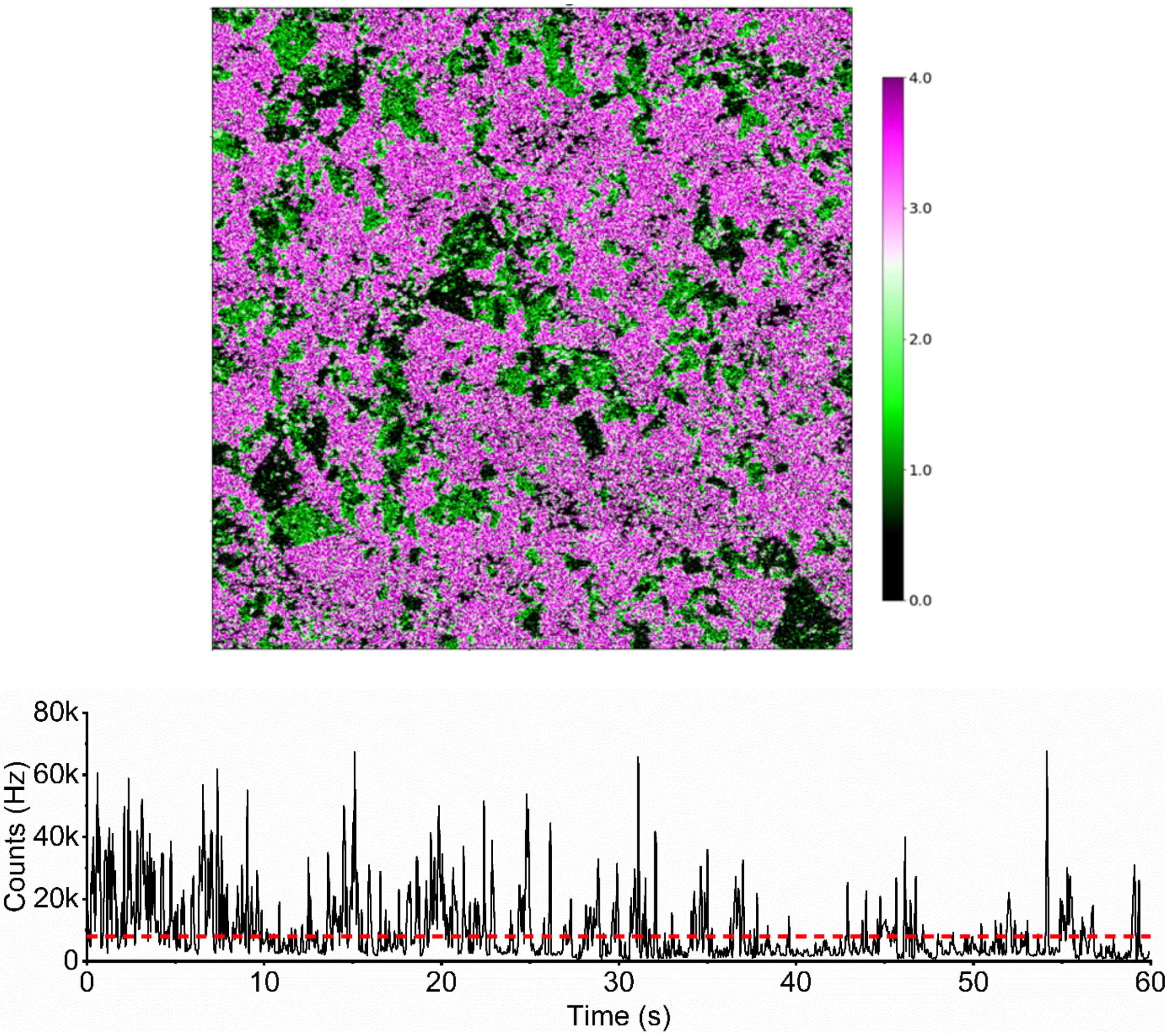

Figure S10. Representative 80 μm × 80 μm FLIM image (above) of dye-labeled SLBs on MXene flaky films on glass (color-coded gradient bar indicates lifetime in ns) (above) and intensity time trace of single-molecule bursts taken at a MXene area (green area) (below). Red dash line represents the cutoff threshold of 400 photons used for lifetime data analysis.

Table S1. Buffers used in this work.

Buffer name	Composition	Use
**PBS-Na⁺**	137 mM NaCl, 2.7 mM KCl, 10 mM Na_2HPO_4, and 1.8 mM KH_2PO_4 supplemented with 1.463 M NaCl and EDTA 2 mM (final pH 6.9)	Specific immobilization of NROs *via gly*-MXene chemistry
**PBS-Na⁺-Tween**	137 mM NaCl, 2.7 mM KCl, 10 mM Na_2HPO_4, and 1.8 mM KH_2PO_4 supplemented with 1.463 M NaCl, EDTA 2 mM and Tween-20 0.005 % (final pH 6.9)	Washing
**PBS 1x**	137 mM NaCl, 2.7 mM KCl, 10 mM Na_2HPO_4, and 1.8 mM KH_2PO_4 pH 7.4	Immobilization of biotinylated NROs on BSA-NeutrAvidin-passivated glass
**TAE-Mg^{2+}-20**	40 mM Tris-acetate, 1 mM EDTA, 20 mM $MgCl_2$ pH 8.0	DNA origami folding
**TAE-Mg^{2+}-12.5**	40 mM Tris-acetate, 1 mM EDTA, 12.5 mM $MgCl_2$ pH 8.0	Adsorption of ssDNA-ATTO542 on MXene and nonspecific adsorption of $NRO_{No\text{-}Gly}$
**LUVs**	5 mM Tris, 1 mM EDTA, 0.5 mM Trolox and 150 mM NaCl at pH 7.0	Dispersing LUVs and incubating/washing SLBs on MXene and glass

Table S2. Sequences of staples $NRO_{Gly\text{-}1nm}$ and $NRO_{Gly\text{-}3nm}$ or 0 bp (Scheme 1, main text)

5'position	Sequence	Modification
10[175]	TTAACGTCTAACATAAAAACAGGTAACGGA	None
23[224]	GCACAGACAATATTTTTGAATGGGGTCAGTA	None
2[175]	TATTAAGAAGCGGGGTTTTGCTCGTAGCAT	None
4[63]	ATAAGGGAACCGGATATTCATTACGTCAGGACGTTGGGAA	None
13[120]	AAAGGCCGGAGACAGCTAGCTGATAAATTAATTTTTGT	None
17[96]	GCTTTCCGATTACGCCAGCTGGCGGCTGTTTC	None
13[96]	TAGGTAAACTATTTTTGAGAGATCAAACGTTA	None
16[207]	ACCTTTTTATTTTAGTTAATTTCATAGGGCTT	None
14[207]	AATTGAGAATTCTGTCCAGACGACTAAACCAA	None
5[32]	CATCAAGTAAAACGAACTAACGAGTTGAGA	None
6[79]	TTATACCACCAAATCAACGTAACGAACGAG	None
15[192]	TCAAATATAACCTCCGGCTTAGGTAACAATTT	None
3[32]	AATACGTTTGAAAGAGGACAGACTGACCTT	None
12[143]	TTCTACTACGCGAGCTGAAAAGGTTACCGCGC	None
18[207]	CGCGCAGATTACCTTTTTTAATGGGAGAGACT	None
4[79]	GCGCAGACAAGAGGCAAAAGAATCCCTCAG	None
20[47]	TTAATGAACTAGAGGATCCCCGGGGGGTAACG	None
2[207]	TTTCGGAAGTGCCGTCGAGAGGGTGAGTTTCG	None
16[271]	CTTAGATTTAAGGCGTTAAATAAAGCCTGT	None
17[160]	AGAAAACAAAGAAGATGATGAAACAGGCTGCG	None

14[143]	CAACCGTTTCAAATCACCATCAATTCGAGCCA	None
1[96]	AAACAGCTTTTTGCGGGATCGTCAACACTAAA	None
19[192]	ATTATACTAAGAAACCACCAGAAGTCAACAGT	None
5[224]	TCAAGTTTCATTAAAGGTGAATATAAAAGA	None
7[128]	AGACGACAAAGAAGTTTTGCCATAATTCGAGCTTCAA	None
12[175]	TTTTATTTAAGCAAATCAGATATTTTTTGT	None
16[111]	TGTAGCCATTAAAATTCGCATTAAATGCCGGA	None
23[96]	CCCGATTTAGAGCTTGACGGGGAAAAAGAATA	None
10[239]	GCCAGTTAGAGGGTAATTGAGCGCTTTAAGAA	None
8[175]	ATACCCAACAGTATGTTAGCAAATTAGAGC	None
19[56]	TACCGAGCTCGAATTCGGGAAACCTGTCGTGCAGCTGATT	None
15[128]	TAAATCAAAATAATTCGCGTCTCGGAAACC	None
9[160]	AGAGAGAAAAAAATGAAAATAGCAAGCAAACT	None
18[175]	CTGAGCAAAAATTAATTACATTTTGGGTTA	None
18[143]	CAACTGTTGCGCCATTCGCCATTCAAACATCA	None
23[160]	TAAAAGGGACATTCTGGCCAACAAAGCATC	None
5[160]	GCAAGGCCTCACCAGTAGCACCATGGGCTTGA	None
15[160]	ATCGCAAGTATGTAAATGCTGATGATAGGAAC	None
3[160]	TTGACAGGCCACCACCAGAGCCGCGATTTGTA	None
7[248]	GTTTATTTTGTCACAATCTTACCGAAGCCCTTTAATATCA	None
11[224]	GCGAACCTCCAAGAACGGGTATGACAATAA	None
4[111]	GACCTGCTCTTTGACCCCCAGCGAGGGAGTTA	None
2[111]	AAGGCCGCTGATACCGATAGTTGCGACGTTAG	None
8[239]	AAGTAAGCAGACACCACGGAATAATATTGACG	None
10[47]	CTGTAGCTTGACTATTATAGTCAGTTCATTGA	None
1[256]	CAGGAGGTGGGGTCAGTGCCTTGAGTCTCTGAATTTACCG	None
20[143]	AAGCCTGGTACGAGCCGGAAGCATAGATGATG	None
22[143]	TCGGCAAATCCTGTTTGATGGTGGACCCTCAA	None
10[79]	GATGGCTTATCAAAAAGATTAAGAGCGTCC	None
1[128]	TGACAACTCGCTGAGGCTTGCATTATACCA	None
12[47]	TAAATCGGGATTCCCAATTCTGCGATATAATG	None
21[192]	TGAAAGGAGCAAATGAAAAATCTAGAGATAGA	None
1[160]	TTAGGATTGGCTGAGACTCCTCAATAACCGAT	None
11[160]	CCAATAGCTCATCGTAGGAATCATGGCATCAA	None
11[64]	GATTTAGTCAATAAAGCCTCAGAGAACCCTCA	None
17[32]	TGCATCTTTCCCAGTCACGACGGCCTGCAG	None
8[111]	AATAGTAAACACTATCATAACCCTCATTGTGA	None
14[175]	CATGTAATAGAATATAAAGTACCAAGCCGT	None
19[248]	CGTAAAACAGAAATAAAAATCCTTTGCCCGAAAGATTAGA	None
20[207]	GCGGAACATCTGAATAATGGAAGGTACAAAAT	None
4[255]	AGCCACCACTGTAGCGCGTTTTCAAGGGAGGGAAGGTAAA	None
18[271]	CTTTTACAAAATCGTCGCTATTAGCGATAG	None
16[143]	GCCATCAAGCTCATTTTTTAACCACAAATCCA	None
10[143]	CCAACAGGAGCGAACCAGACCGGAGCCTTTAC	None
22[271]	CAGAAGATTAGATAATACATTTGTCGACAA	None
16[47]	ACAAACGGAAAAGCCCCAAAAACACTGGAGCA	None
16[79]	GCGAGTAAAAATATTTAAATTGTTACAAAG	None

20[239]	ATTTTAAAATCAAAATTATTGCACGGATTCG	None
14[239]	AGTATAAAGTTCAGCTAATGCAGATGTCTTTC	None
0[79]	ACAACTTTCAACAGTTTCAGCGGATGTATCGG	None
4[143]	TCATCGCCAACAAAGTACAACGGACGCCAGCA	None
7[56]	ATGCAGATACATAACGGGAATCGTCATAAATAAAGCAAAG	None
17[224]	CATAAATCTTTGAATACCAAGTGTTAGAAC	None
6[175]	CAGCAAAAGGAAACGTCACCAATGAGCCGC	None
14[111]	GAGGGTAGGATTCAAAAGGGTGAGACATCCAA	None
15[32]	TAATCAGCGGATTGACCGTAATCGTAACCG	None
6[111]	ATTACCTTTGAATAAGGCTTGCCCAAATCCGC	None
8[207]	AAGGAAACATAAAGGTGGCAACATTATCACCG	None
12[239]	CTTATCATTCCCGACTTGCGGGAGCCTAATTT	None
20[79]	TTCCAGTCGTAATCATGGTCATAAAAGGGG	None
7[192]	ATACATACCGAGGAAACGCAATAAGAAGCGCATTAGACGG	None
19[128]	CACAACAGGTGCCTAATGAGTGCCCAGCAG	None
13[64]	TATATTTTGTCATTGCCTGAGAGTGGAAGATTGTATAAGC	None
16[63]	CGGATTCTGACGACAGTATCGGCCGCAAGGCGATTAAGTT	None
5[192]	CGATAGCATTGAGCCATTTGGGAACGTAGAAA	None
13[160]	GTAATAAGTTAGGCAGAGGCATTTATGATATT	None
6[271]	ACCGATTGTCGGCATTTTCGGTCATAATCA	None
22[175]	ACCTTGCTTGGTCAGTTGGCAAAGAGCGGA	None
3[192]	GGCCTTGAAGAGCCACCACCCTCAGAAACCAT	None
21[224]	CTTTAGGGCCTGCAACAGTGCCAATACGTG	None
4[271]	AAATCACCTTCCAGTAAGCGTCAGTAATAA	None
6[239]	GAAATTATTGCCTTTAGCGTCAGACCGGAACC	None
6[47]	TACGTTAAAGTAATCTTGACAAGAACCGAACT	None
23[128]	AACGTGGCGAGAAAGGAAGGGAAACCAGTAA	None
4[175]	CACCAGAAAGGTTGAGGCAGGTCATGAAAG	None
23[192]	ACCCTTCTGACCTGAAAGCGTAAGACGCTGAG	None
17[128]	AGGCAAAGGGAAGGGCGATCGGCAATTCCA	None
23[256]	CTTTAATGCGCGAACTGATAGCCCCACCAG	None
10[111]	TTGCTCCTTTCAAATATCGCGTTTGAGGGGGT	None
22[207]	AGCCAGCAATTGAGGAAGGTTATCATCATTTT	None
22[79]	TGGAACAACCGCCTGGCCCTGAGGCCCGCT	None
1[192]	GCGGATAACCTATTATTCTGAAACAGACGATT	None
0[111]	TAAATGAATTTTCTGTATGGGATTAATTTCTT	None
1[64]	TTTATCAGGACAGCATCGGAACGACACCAACCTAAAACGA	None
3[96]	ACACTCATCCATGTTACTTAGCCGAAAGCTGC	None
6[207]	TCACCGACGCACCGTAATCAGTAGCAGAACCG	None
19[96]	CTGTGTGATTGCGTTGCGCTCACTAGAGTTGC	None
19[160]	GCAATTCACATATTCCTGATTATCAAAGTGTA	None
12[111]	TAAATCATATAACCTGTTTAGCTAACCTTTAA	None
20[175]	ATTATCATTCAATATAATCCTGACAATTAC	None
22[239]	TTAACACCAGCACTAACAACTAATCGTTATTA	None
10[207]	ATCCCAATGAGAATTAACTGAACAGTTACCAG	None
15[96]	ATATTTTGGCTTTCATCAACATTATCCAGCCA	None
15[224]	CCTAAATCAAAATCATAGGTCTAAACAGTA	None

20[111]	CACATTAAAATTGTTATCCGCTCATGCGGGCC	None
22[111]	GCCCGAGAGTCCACGCTGGTTTGCAGCTAACT	None
19[224]	CTACCATAGTTTGAGTAACATTTAAAATAT	None
10[191]	GAAACGATAGAAGGCTTATCCGGTCTCATCGAGAACAAGC	None
2[143]	ATATTCGGAACCATCGCCCACGCAGAGAAGGA	None
21[160]	TCAATATCGAACCTCAAATATCAATTCCGAAA	None
7[96]	TAAGAGCAAATGTTTAGACTGGATAGGAAGCC	None
13[224]	ACAACATGCCAACGCTCAACAGTCTTCTGA	None
11[96]	AATGGTCAACAGGCAAGGCAAAGAGTAATGTG	None
23[64]	AAAGCACTAAATCGGAACCCTAATCCAGTT	None
2[47]	ACGGCTACAAAAGGAGCCTTTAATGTGAGAAT	None
16[175]	TATAACTAACAAAGAACGCGAGAACGCCAA	None
0[207]	TCACCAGTACAAACTACAACGCCTAGTACCAG	None
21[128]	GCGAAAAATCCCTTATAAATCAAGCCGGCG	None
21[96]	AGCAAGCGTAGGGTTGAGTGTTGTAGGGAGCC	None
14[79]	GCTATCAGAAATGCAATGCCTGAATTAGCA	None
9[96]	CGAAAGACTTTGATAAGAGGTCATATTTCGCA	None
13[184]	GACAAAAGGTAAAGTAATCGCCATATTTAACAAAACTTTT	None
18[79]	GATGTGCTTCAGGAAGATCGCACAATGTGA	None
1[32]	AGGCTCCAGAGGCTTTGAGGACACGGGTAA	None
12[79]	AAATTAAGTTGACCATTAGATACTTTTGCG	None
18[111]	TCTTCGCTGCACCGCTTCTGGTGCGGCCTTCC	None
1[224]	GTATAGCAAACAGTTAATGCCCAATCCTCA	None
3[128]	AGCGCGATGATAAATTGTGTCGTGACGAGA	None
8[79]	AATACTGCCCAAAAGGAATTACGTGGCTCA	None
5[128]	AACACCAAATTTCAACTTTAATCGTTTACC	None
16[239]	GAATTTATTTAATGGTTTGAAATATTCTTACC	None
5[96]	TCATTCAGATGCGATTTTAAGAACAGGCATAG	None
8[271]	AATAGCTATCAATAGAAAATTCAACATTCA	None
3[224]	TTAAAGCCAGAGCCGCCACCCTCGACAGAA	None
21[64]	GCCCTTCAGAGTCCACTATTAAAGGGTGCCGT	None
13[256]	GTTTATCAATATGCGTTATACAAACCGACCGTGTGATAAA	None
16[255]	GAGAAGAGATAACCTTGCTTCTGTTCGGGAGAAACAATAA	None
20[271]	CTCGTATTAGAAATTGCGTAGATACAGTAC	None
12[207]	GTACCGCAATTCTAAGAACGCGAGTATTATTT	None
8[47]	ATCCCCCTATACCACATTCAACTAGAAAAATC	None
8[143]	CTTTTGCAGATAAAAACCAAAATAAAGACTCC	None
4[207]	CCACCCTCTATTCACAAACAAATACCTGCCTA	None
7[224]	AACGCAAAGATAGCCGAACAAACCCTGAAC	None
6[143]	GATGGTTTGAACGAGTAGTAAATTTACCATTA	None
2[79]	CAGCGAAACTTGCTTTCGAGGTGTTGCTAA	None
17[192]	CATTTGAAGGCGAATTATTCATTTTTGTTTGG	None
21[256]	GCCGTCAAAAAACAGAGGTGAGGCCTATTAGT	None
0[271]	CCACCCTCATTTTCAGGGATAGCAACCGTACT	None
11[256]	GCCTTAAACCAATCAATAATCGGCACGCGCCT	None
21[32]	TTTTCACTCAAAGGGCGAAAAACCATCACC	None
0[175]	TCCACAGACAGCCCTCATAGTTAGCGTAACGA	None

10[271]	ACGCTAACACCCACAAGAATTGAAAATAGC	None
23[32]	CAAATCAAGTTTTTGGGGTCGAAACGTGGA	None
2[239]	GCCCGTATCCGGAATAGGTGTATCAGCCCAAT	None
12[271]	TGTAGAAATCAAGATTAGTTGCTCTTACCA	None
14[271]	TTAGTATCACAATAGATAAGTCCACGAGCA	None
11[32]	AACAGTTTTGTACCAAAAACATTTTATTTC	None
0[143]	TCTAAAGTTTTGTCGTCTTTCCAGCCGACAA	None
9[256]	GAGAGATAGAGCGTCTTTCCAGAGGTTTTGAA	None
19[32]	GTCGACTTCGGCCAACGCGCGGGGTTTTTC	None
0[47]	AGAAAGGAACAACTAAAGGAATTCAAAAAAA	None
2[271]	GTTTTAACTTAGTACCGCCACCCAGAGCCA	None
0[239]	AGGAACCCATGTACCGTAACACTTGATATAA	None
13[32]	AACGCAAAATCGATGAACGGTACCGGTTGA	None
14[47]	AACAAGAGGGATAAAAATTTTTAGCATAAAGC	None
22[47]	CTCCAACGCAGTGAGACGGGCAACCAGCTGCA	None
9[32]	TTTACCCCAACATGTTTTAAATTTCCATAT	None
7[32]	TTTAGGACAAATGCTTTAAACAATCAGGTC	None
9[224]	AAAGTCACAAAATAAACAGCCAGCGTTTTA	5‘-triglycine
9[64]	CGGATTGCAGAGCTTAATTGCTGAAACGAGTA	5‘-triglycine
4[239]	GCCTCCCTCAGAATGGAAAGCGCAGTAACAGT	5‘-triglycine
18[47]	CCAGGGTTGCCAGTTTGAGGGGACCCGTGGGA	5‘-triglycine
18[239]	CCTGATTGCAATATATGTGAGTGATCAATAGT	5‘-triglycine
4[47]	GACCAACTAATGCCACTACGAAGGGGGTAGCA	5‘-triglycine
7[160]	TTATTACGAAGAACTGGCATGATTGCGAGAGG	None
10[127]	TAGAGAGTTATTTTCATTTGGGGATAGTAGTAGCATTA	5’ATTO542 ($NRO_{Gly\text{-}3nm}$)

Table S3. Sequences of staples for NRO structures with protrusions (See table S3 for sequences of protruding strands)

5’position	Sequence	Modification
21[160]	TCAATATCGAACCTCAAATATCAATTCCGAAA	None
10[239]	GCCAGTTAGAGGGTAATTGAGCGCTTTAAGAA	None
2[47]	ACGGCTACAAAAGGAGCCTTTAATGTGAGAAT	None
19[192]	ATTATACTAAGAAACCACCAGAAGTCAACAGT	None
12[207]	GTACCGCAATTCTAAGAACGCGAGTATTATTT	None
6[239]	GAAATTATTGCCTTTAGCGTCAGACCGGAACC	None
22[271]	CAGAAGATTAGATAATACATTTGTCGACAA	None
3[96]	ACACTCATCCATGTTACTTAGCCGAAAGCTGC	None
17[128]	AGGCAAAGGGAAGGGCGATCGGCAATTCCA	None
15[128]	TAAATCAAAATAATTCGCGTCTCGGAAACC	None
18[175]	CTGAGCAAAAATTAATTACATTTTGGGTTA	None
19[160]	GCAATTCACATATTCCTGATTATCAAAGTGTA	None
0[111]	TAAATGAATTTTCTGTATGGGATTAATTTCTT	None
5[128]	AACACCAAATTTCAACTTTAATCGTTTACC	None
12[143]	TTCTACTACGCGAGCTGAAAAGGTTACCGCGC	None
16[175]	TATAACTAACAAAGAACGCGAGAACGCCAA	None

22[111]	GCCCGAGAGTCCACGCTGGTTTGCAGCTAACT	None
19[224]	CTACCATAGTTTGAGTAACATTTAAAATAT	None
2[79]	CAGCGAAACTTGCTTTCGAGGTGTTGCTAA	None
17[224]	CATAAATCTTTGAATACCAAGTGTTAGAAC	None
17[96]	GCTTTCCGATTACGCCAGCTGGCGGCTGTTTC	None
19[96]	CTGTGTGATTGCGTTGCGCTCACTAGAGTTGC	None
16[255]	GAGAAGAGATAACCTTGCTTCTGTTCGGGAGAAACAATAA	None
5[160]	GCAAGGCCTCACCAGTAGCACCATGGGCTTGA	None
4[63]	ATAAGGGAACCGGATATTCATTACGTCAGGACGTTGGGAA	None
5[224]	TCAAGTTTCATTAAAGGTGAATATAAAAGA	None
5[96]	TCATTCAGATGCGATTTTAAGAACAGGCATAG	None
12[47]	TAAATCGGGATTCCCAATTCTGCGATATAATG	None
22[175]	ACCTTGCTTGGTCAGTTGGCAAAGAGCGGA	None
16[271]	CTTAGATTTAAGGCGTTAAATAAAGCCTGT	None
4[271]	AAATCACCTTCCAGTAAGCGTCAGTAATAA	None
15[160]	ATCGCAAGTATGTAAATGCTGATGATAGGAAC	None
22[239]	TTAACACCAGCACTAACAACTAATCGTTATTA	None
4[207]	CCACCCTCTATTCACAAACAAATACCTGCCTA	None
7[56]	ATGCAGATACATAACGGGAATCGTCATAAATAAAGCAAAG	None
4[79]	GCGCAGACAAGAGGCAAAAGAATCCCTCAG	None
3[224]	TTAAAGCCAGAGCCGCCACCCTCGACAGAA	None
16[63]	CGGATTCTGACGACAGTATCGGCCGCAAGGCGATTAAGTT	None
20[239]	ATTTTAAAATCAAAATTATTTGCACGGATTCG	None
7[128]	AGACGACAAAGAAGTTTTGCCATAATTCGAGCTTCAA	None
16[111]	TGTAGCCATTAAAATTCGCATTAAATGCCGGA	None
11[64]	GATTTAGTCAATAAAGCCTCAGAGAACCCTCA	None
13[64]	TATATTTTGTCATTGCCTGAGAGTGGAAGATTGTATAAGC	None
23[64]	AAAGCACTAAATCGGAACCCTAATCCAGTT	None
2[175]	TATTAAGAAGCGGGGTTTTGCTCGTAGCAT	None
15[32]	TAATCAGCGGATTGACCGTAATCGTAACCG	None
1[64]	TTTATCAGGACAGCATCGGAACGACACCAACCTAAAACGA	None
4[111]	GACCTGCTCTTTGACCCCCAGCGAGGGAGTTA	None
7[248]	GTTTATTTTGTCACAATCTTACCGAAGCCCTTTAATATCA	None
14[79]	GCTATCAGAAATGCAATGCCTGAATTAGCA	None
14[207]	AATTGAGAATTCTGTCCAGACGACTAAACCAA	None
13[224]	ACAACATGCCAACGCTCAACAGTCTTCTGA	None
19[56]	TACCGAGCTCGAATTCGGGAAACCTGTCGTGCAGCTGATT	None
1[256]	CAGGAGGTGGGGTCAGTGCCTTGAGTCTCTGAATTTACCG	None
11[224]	GCGAACCTCCAAGAACGGGTATGACAATAA	None
1[128]	TGACAACTCGCTGAGGCTTGCATTATACCA	None
21[192]	TGAAAGGAGCAAATGAAAAATCTAGAGATAGA	None
5[192]	CGATAGCATTGAGCCATTTGGGAACGTAGAAA	None
13[160]	GTAATAAGTTAGGCAGAGGCATTTATGATATT	None
1[192]	GCGGATAACCTATTATTCTGAAACAGACGATT	None
10[175]	TTAACGTCTAACATAAAAACAGGTAACGGA	None
15[96]	ATATTTGGCTTTCATCAACATTATCCAGCCA	None
16[239]	GAATTTATTTAATGGTTTGAAATATTCTTACC	None

3[128]	AGCGCGATGATAAATTGTGTCGTGACGAGA	None
12[111]	TAAATCATATAACCTGTTTAGCTAACCTTTAA	None
21[224]	CTTTAGGGCCTGCAACAGTGCCAATACGTG	None
17[32]	TGCATCTTTCCCAGTCACGACGGCCTGCAG	None
23[128]	AACGTGGCGAGAAAGGAAGGGAAACCAGTAA	None
21[96]	AGCAAGCGTAGGGTTGAGTGTTGTAGGGAGCC	None
1[224]	GTATAGCAAACAGTTAATGCCCAATCCTCA	None
19[128]	CACAACAGGTGCCTAATGAGTGCCCAGCAG	None
8[207]	AAGGAAACATAAAGGTGGCAACATTATCACCG	None
20[47]	TTAATGAACTAGAGGATCCCCGGGGGGTAACG	None
6[207]	TCACCGACGCACCGTAATCAGTAGCAGAACCG	None
21[256]	GCCGTCAAAAAACAGAGGTGAGGCCTATTAGT	None
9[96]	CGAAAGACTTTGATAAGAGGTCATATTTCGCA	None
3[192]	GGCCTTGAAGAGCCACCACCCTCAGAAACCAT	None
23[160]	TAAAAGGGACATTCTGGCCAACAAAGCATC	None
14[175]	CATGTAATAGAATATAAAGTACCAAGCCGT	None
11[160]	CCAATAGCTCATCGTAGGAATCATGGCATCAA	None
6[271]	ACCGATTGTCGGCATTTTCGGTCATAATCA	None
21[128]	GCGAAAAATCCCTTATAAATCAAGCCGGCG	None
22[143]	TCGGCAAATCCTGTTTGATGGTGGACCCTCAA	None
4[143]	TCATCGCCAACAAAGTACAACGGACGCCAGCA	None
8[47]	ATCCCCCTATACCACATTCAACTAGAAAAATC	None
22[207]	AGCCAGCAATTGAGGAAGGTTATCATCATTTT	None
22[79]	TGGAACAACCGCCTGGCCCTGAGGCCCGCT	None
20[79]	TTCCAGTCGTAATCATGGTCATAAAAGGGG	None
7[96]	TAAGAGCAAATGTTTAGACTGGATAGGAAGCC	None
18[79]	GATGTGCTTCAGGAAGATCGCACAATGTGA	None
17[160]	AGAAAACAAAGAAGATGATGAAACAGGCTGCG	None
11[96]	AATGGTCAACAGGCAAGGCAAAGAGTAATGTG	None
13[96]	TAGGTAAACTATTTTTGAGAGATCAAACGTTA	None
21[64]	GCCCTTCAGAGTCCACTATTAAAGGGTGCCGT	None
7[192]	ATACATACCGAGGAAACGCAATAAGAAGCGCATTAGACGG	None
8[271]	AATAGCTATCAATAGAAAATTCAACATTCA	None
12[175]	TTTTATTTAAGCAAATCAGATATTTTTTGT	None
19[248]	CGTAAAACAGAAATAAAAATCCTTTGCCCGAAAGATTAGA	None
6[143]	GATGGTTTGAACGAGTAGTAAATTTACCATTA	None
12[239]	CTTATCATTCCCGACTTGCGGGAGCCTAATTT	None
6[47]	TACGTTAAAGTAATCTTGACAAGAACCGAACT	None
20[207]	GCGGAACATCTGAATAATGGAAGGTACAAAAT	None
16[143]	GCCATCAAGCTCATTTTTTAACCACAAATCCA	None
10[127]	TAGAGAGTTATTTTCATTTGGGGATAGTAGTAGCATTA	None
18[207]	CGCGCAGATTACCTTTTTTAATGGGAGAGACT	None
20[143]	AAGCCTGGTACGAGCCGGAAGCATAGATGATG	None
10[79]	GATGGCTTATCAAAAAGATTAAGAGCGTCC	None
18[143]	CAACTGTTGCGCCATTCGCCATTCAAACATCA	None
1[32]	AGGCTCCAGAGGCTTTGAGGACACGGGTAA	None
5[32]	CATCAAGTAAAACGAACTAACGAGTTGAGA	None

10[47]	CTGTAGCTTGACTATTATAGTCAGTTCATTGA	None
15[224]	CCTAAATCAAAATCATAGGTCTAAACAGTA	None
20[111]	CACATTAAAATTGTTATCCGCTCATGCGGGCC	None
6[175]	CAGCAAAAGGAAACGTCACCAATGAGCCGC	None
8[175]	ATACCCAACAGTATGTTAGCAAATTAGAGC	None
10[191]	GAAACGATAGAAGGCTTATCCGGTCTCATCGAGAACAAGC	None
23[256]	CTTTAATGCGCGAACTGATAGCCCCACCAG	None
2[143]	ATATTCGGAACCATCGCCCACGCAGAGAAGGA	None
10[111]	TTGCTCCTTTCAAATATCGCGTTTGAGGGGGT	None
14[111]	GAGGGTAGGATTCAAAAGGGTGAGACATCCAA	None
8[239]	AAGTAAGCAGACACCACGGAATAATATTGACG	None
20[271]	CTCGTATTAGAAATTGCGTAGATACAGTAC	None
8[111]	AATAGTAAACACTATCATAACCCTCATTGTGA	None
15[192]	TCAAATATAACCTCCGGCTTAGGTAACAATTT	None
8[79]	AATACTGCCCAAAAGGAATTACGTGGCTCA	None
16[79]	GCGAGTAAAAATATTTAAATTGTTACAAAG	None
16[207]	ACCTTTTTATTTTAGTTAATTTCATAGGGCTT	None
14[239]	AGTATAAAGTTCAGCTAATGCAGATGTCTTTC	None
10[207]	ATCCCAATGAGAATTAACTGAACAGTTACCAG	None
12[79]	AAATTAAGTTGACCATTAGATACTTTTGCG	None
18[271]	CTTTTACAAAATCGTCGCTATTAGCGATAG	None
0[79]	ACAACTTTCAACAGTTTCAGCGGATGTATCGG	None
20[175]	ATTATCATTCAATATAATCCTGACAATTAC	None
1[160]	TTAGGATTGGCTGAGACTCCTCAATAACCGAT	None
13[120]	AAAGGCCGGAGACAGCTAGCTGATAAATTAATTTTTGT	None
3[32]	AATACGTTTGAAAGAGGACAGACTGACCTT	None
0[207]	TCACCAGTACAAACTACAACGCCTAGTACCAG	None
13[184]	GACAAAAGGTAAAGTAATCGCCATATTTAACAAAACTTTT	None
23[192]	ACCCTTCTGACCTGAAAGCGTAAGACGCTGAG	None
18[111]	TCTTCGCTGCACCGCTTCTGGTGCGGCCTTCC	None
1[96]	AAACAGCTTTTTGCGGGATCGTCAACACTAAA	None
4[175]	CACCAGAAAGGTTGAGGCAGGTCATGAAAG	None
13[256]	GTTTATCAATATGCGTTATACAAACCGACCGTGTGATAAA	None
3[160]	TTGACAGGCCACCACCAGAGCCGCGATTTGTA	None
23[96]	CCCGATTTAGAGCTTGACGGGGAAAAAGAATA	None
7[224]	AACGCAAAGATAGCCGAACAAACCCTGAAC	None
17[192]	CATTTGAAGGCGAATTATTCATTTTTGTTTGG	None
6[111]	ATTACCTTTGAATAAGGCTTGCCCAAATCCGC	None
4[255]	AGCCACCACTGTAGCGCGTTTTCAAGGGAGGGAAGGTAAA	None
23[224]	GCACAGACAATATTTTTGAATGGGGTCAGTA	None
2[111]	AAGGCCGCTGATACCGATAGTTGCGACGTTAG	None
16[47]	ACAAACGGAAAAGCCCCAAAAACACTGGAGCA	None
6[79]	TTATACCACCAAATCAACGTAACGAACGAG	None
14[143]	CAACCGTTTCAAATCACCATCAATTCGAGCCA	None
2[207]	TTTCGGAAGTGCCGTCGAGAGGGTGAGTTTCG	None
0[239]	AGGAACCCATGTACCGTAACACTTGATATAA	None
11[256]	GCCTTAAACCAATCAATAATCGGCACGCGCCT	None

0[47]	AGAAAGGAACAACTAAAGGAATTCAAAAAAA	None
2[239]	GCCCGTATCCGGAATAGGTGTATCAGCCCAAT	None
14[271]	TTAGTATCACAATAGATAAGTCCACGAGCA	None
12[271]	TGTAGAAATCAAGATTAGTTGCTCTTACCA	None
19[32]	GTCGACTTCGGCCAACGCGCGGGGTTTTTC	None
11[32]	AACAGTTTTGTACCAAAAACATTTTATTTC	None
0[143]	TCTAAAGTTTTGTCGTCTTTCCAGCCGACAA	None
10[271]	ACGCTAACACCCACAAGAATTGAAAATAGC	None
21[32]	TTTTCACTCAAAGGGCGAAAAACCATCACC	None
9[32]	TTTACCCCAACATGTTTTAAATTTCCATAT	None
22[47]	CTCCAACGCAGTGAGACGGGCAACCAGCTGCA	None
9[256]	GAGAGATAGAGCGTCTTTCCAGAGGTTTTGAA	None
0[271]	CCACCCTCATTTTCAGGGATAGCAACCGTACT	None
2[271]	GTTTTAACTTAGTACCGCCACCCAGAGCCA	None
13[32]	AACGCAAAATCGATGAACGGTACCGGTTGA	None
0[175]	TCCACAGACAGCCCTCATAGTTAGCGTAACGA	None
7[32]	TTTAGGACAAATGCTTTAAACAATCAGGTC	None
14[47]	AACAAGAGGGATAAAAATTTTTAGCATAAAGC	None
23[32]	CAAATCAAGTTTTTTGGGGTCGAAACGTGGA	None
8[143]	CTTTTGCAGATAAAAACCAAAATAAAGACTCC	None
7[160]	TTATTACGAAGAACTGGCATGATTGCGAGAGG	None
18[239]	CCTGATTGCAATATATGTGAGTGATCAATAGT	5‘-triglycine
9[64]	CGGATTGCAGAGCTTAATTGCTGAAACGAGTA	5‘-triglycine
4[239]	GCCTCCCTCAGAATGGAAAGCGCAGTAACAGT	5‘-triglycine
9[224]	AAAGTCACAAAATAAACAGCCAGCGTTTTA	5‘-triglycine
4[47]	GACCAACTAATGCCACTACGAAGGGGGTAGCA	5‘-triglycine
18[47]	CCAGGGTTGCCAGTTTGAGGGGACCCGTGGGA	5‘-triglycine

Table S4. Sequences of 3-20 bp protrusions for NRO nanopositioners ≥4 nm

5’position	Sequence	Modification
10[149]	CGCCAAACTCCAACAGGAGCGAACCAGACCGGAGCC	5’ATTO542-3-bp protrusion (4 nm)
9[155]	TTTACAGAGAGAAAAAAATGAAAATAGCAAGGCG	Unmodified hybridizing 3-nt (4 nm)
10[149]	CGCCGCCAAACTCCAACAGGAGCGAACCAGACCGGAGCC	5’ATTO542-6-bp protrusion (5 nm)
9[155]	TTTACAGAGAGAAAAAAATGAAAATAGCAAGGCGGCG	Unmodified hybridizing 6-nt (5 nm)
9[155]	TTTACAGAGAGAAAAAAATGAAAATAGCAAG ATTCGTTGCATAGTGCATAT	Unmodified hybridizing 20-nt (10 nm)
External	ATATGCACTATGCAACGAAT	5‘-ATTO542-20-bp protrusion (10 nm)

Table S5. p7249 scaffold sequence (5’ to 3’)

TTCCCTTCCTTTCTCGCCACGTTCGCCGGCTTTCCCCGTCAAGCTCTAAATCGGGGGCTCCCTTTAGGGTTCCGATTTAGTGCTT
TACGGCACCTCGACCCCAAAAAACTTGATTTGGGTGATGGTTCACGTAGTGGGCCATCGCCCTGATAGACGGTTTTTCGCCCTT
TGACGTTGGAGTCCACGTTCTTTAATAGTGGACTCTTGTTCCAAACTGGAACAACACTCAACCCTATCTCGGGCTATTCTTTTGA

TTTATAAGGGATTTTGCCGATTTCGGAACCACCATCAAACAGGATTTTCGCCTGCTGGGGCAAACCAGCGTGGACCGCTTGCTG
CAACTCTCTCAGGGCCAGGCGGTGAAGGGCAATCAGCTGTTGCCCGTCTCACTGGTGAAAAGAAAAACCACCCTGGCGCCCA
ATACGCAAACCGCCTCTCCCCGCGCGTTGGCCGATTCATTAATGCAGCTGGCACGACAGGTTTCCCGACTGGAAAGCGGGCAG
TGAGCGCAACGCAATTAATGTGAGTTAGCTCACTCATTAGGCACCCCAGGCTTTACACTTTATGCTTCCGGCTCGTATGTTGTGT
GGAATTGTGAGCGGATAACAATTTCACACAGGAAACAGCTATGACCATGATTACGAATTCGAGCTCGGTACCCGGGGATCCTCT
AGAGTCGACCTGCAGGCATGCAAGCTTGGCACTGGCCGTCGTTTTACAACGTCGTGACTGGGAAAACCCTGGCGTTACCCAAC
TTAATCGCCTTGCAGCACATCCCCCTTTCGCCAGCTGGCGTAATAGCGAAGAGGCCCGCACCGATCGCCCTTCCCAACAGTTGC
GCAGCCTGAATGGCGAATGGCGCTTTGCCTGGTTTCCGGCACCAGAAGCGGTGCCGGAAAGCTGGCTGGAGTGCGATCTTCCT
GAGGCCGATACTGTCGTCGTCCCCTCAAACTGGCAGATGCACGGTTACGATGCGCCCATCTACACCAACGTGACCTATCCCATT
ACGGTCAATCCGCCGTTTGTTCCCACGGAGAATCCGACGGGTTGTTACTCGCTCACATTTAATGTTGATGAAAGCTGGCTACAG
GAAGGCCAGACGCGAATTATTTTTGATGGCGTTCCTATTGGTTAAAAAATGAGCTGATTTAACAAAAATTTAATGCGAATTTTAA
CAAAATATTAACGTTTACAATTTAAATATTTGCTTATACAATCTTCCTGTTTTTGGGGCTTTTCTGATTATCAACCGGGGTACATAT
GATTGACATGCTAGTTTTACGATTACCGTTCATCGATTCTCTTGTTTGCTCCAGACTCTCAGGCAATGACCTGATAGCCTTTGTAG
ATCTCTCAAAAATAGCTACCCTCTCCGGCATTAATTTATCAGCTAGAACGGTTGAATATCATATTGATGGTGATTTGACTGTCTCC
GGCCTTTCTCACCCTTTTGAATCTTTACCTACACATTACTCAGGCATTGCATTTAAAATATATGAGGGTTCTAAAAATTTTTATCCT
TGCGTTGAAATAAAGGCTTCTCCCGCAAAAGTATTACAGGGTCATAATGTTTTTGGTACAACCGATTTAGCTTTATGCTCTGAGG
CTTTATTGCTTAATTTTGCTAATTCTTTGCCTTGCCTGTATGATTTATTGGATGTTAATGCTACTACTATTAGTAGAATTGATGCCAC
CTTTTCAGCTCGCGCCCCAAATGAAAATATAGCTAAACAGGTTATTGACCATTTGCGAAATGTATCTAATGGTCAAACTAAATCTA
CTCGTTCGCAGAATTGGGAATCAACTGTTATATGGAATGAAACTTCCAGACACCGTACTTTAGTTGCATATTTAAAACATGTTGA
GCTACAGCATTATATTCAGCAATTAAGCTCTAAGCCATCCGCAAAAATGACCTCTTATCAAAAGGAGCAATTAAAGGTACTCTCT
AATCCTGACCTGTTGGAGTTTGCTTCCGGTCTGGTTCGCTTTGAAGCTCGAATTAAAACGCGATATTTGAAGTCTTTCGGGCTTC
CTCTTAATCTTTTTGATGCAATCCGCTTTGCTTCTGACTATAATAGTCAGGGTAAAGACCTGATTTTTGATTTATGGTCATTCTCGT
TTTCTGAACTGTTTAAAGCATTTGAGGGGGATTCAATGAATATTTATGACGATTCCGCAGTATTGGACGCTATCCAGTCTAAACAT
TTTACTATTACCCCCTCTGGCAAAACTTCTTTTGCAAAAGCCTCTCGCTATTTTGGTTTTTATCGTCGTCTGGTAAACGAGGGTTA
TGATAGTGTTGCTCTTACTATGCCTCGTAATTCCTTTTGGCGTTATGTATCTGCATTAGTTGAATGTGGTATTCCTAAATCTCAACT
GATGAATCTTTCTACCTGTAATAATGTTGTTCCGTTAGTTCGTTTTATTAACGTAGATTTTTCTTCCCAACGTCCTGACTGGTATAA
TGAGCCAGTTCTTAAAATCGCATAAGGTAATTCACAATGATTAAAGTTGAAATTAAACCATCTCAAGCCCAATTTACTACTCGTT
CTGGTGTTTCTCGTCAGGGCAAGCCTTATTCACTGAATGAGCAGCTTTGTTACGTTGATTTGGGTAATGAATATCCGGTTCTTGTC
AAGATTACTCTTGATGAAGGTCAGCCAGCCTATGCGCCTGGTCTGTACACCGTTCATCTGTCCTCTTTCAAAGTTGGTCAGTTCG
GTTCCCTTATGATTGACCGTCTGCGCCTCGTTCCGGCTAAGTAACATGGAGCAGGTCGCGGATTTCGACACAATTTATCAGGCGA
TGATACAAATCTCCGTTGTACTTTGTTTCGCGCTTGGTATAATCGCTGGGGGTCAAAGATGAGTGTTTTAGTGTATTCTTTTGCCT
CTTTCGTTTTAGGTTGGTGCCTTCGTAGTGGCATTACGTATTTTACCCGTTTAATGGAAACTTCCTCATGAAAAAGTCTTTAGTCC
TCAAAGCCTCTGTAGCCGTTGCTACCCTCGTTCCGATGCTGTCTTTCGCTGCTGAGGGTGACGATCCCGCAAAAGCGGCCTTTA
ACTCCCTGCAAGCCTCAGCGACCGAATATATCGGTTATGCGTGGGCGATGGTTGTTGTCATTGTCGGCGCAACTATCGGTATCAA
GCTGTTTAAGAAATTCACCTCGAAAGCAAGCTGATAAACCGATACAATTAAAGGCTCCTTTTGGAGCCTTTTTTTTGGAGATTTT
CAACGTGAAAAAATTATTATTCGCAATTCCTTTAGTTGTTCCTTTCTATTCTCACTCCGCTGAAACTGTTGAAAGTTGTTTAGCAA
AATCCCATACAGAAAATTCATTTACTAACGTCTGGAAAGACGACAAAACTTTAGATCGTTACGCTAACTATGAGGGCTGTCTGTG
GAATGCTACAGGCGTTGTAGTTTGTACTGGTGACGAAACTCAGTGTTACGGTACATGGGTTCCTATTGGGCTTGCTATCCCTGAA
AATGAGGGTGGTGGCTCTGAGGGTGGCGGTTCTGAGGGTGGCGGTTCTGAGGGTGGCGGTACTAAACCTCCTGAGTACGGTGA
TACACCTATTCCGGGCTATACTTATATCAACCCTCTCGACGGCACTTATCCGCCTGGTACTGAGCAAAACCCCGCTAATCCTAATC
CTTCTCTTGAGGAGTCTCAGCCTCTTAATACTTTCATGTTTCAGAATAATAGGTTCCGAAATAGGCAGGGGGCATTAACTGTTTAT
ACGGGCACTGTTACTCAAGGCACTGACCCCGTTAAAACTTATTACCAGTACACTCCTGTATCATCAAAAGCCATGTATGACGCTT
ACTGGAACGGTAAATTCAGAGACTGCGCTTTCCATTCTGGCTTTAATGAGGATTTATTTGTTTGTGAATATCAAGGCCAATCGTC
TGACCTGCCTCAACCTCCTGTCAATGCTGGCGGCGGCTCTGGTGGTGGTTCTGGTGGCGGCTCTGAGGGTGGTGGCTCTGAGG
GTGGCGGTTCTGAGGGTGGCGGCTCTGAGGGAGGCGGTTCCGGTGGTGGCTCTGGTTCCGGTGATTTTGATTATGAAAAGATG
GCAAACGCTAATAAGGGGGCTATGACCGAAAATGCCGATGAAAACGCGCTACAGTCTGACGCTAAAGGCAAACTTGATTCTGT
CGCTACTGATTACGGTGCTGCTATCGATGGTTTCATTGGTGACGTTTCCGGCCTTGCTAATGGTAATGGTGCTACTGGTGATTTTG
CTGGCTCTAATTCCCAAATGGCTCAAGTCGGTGACGGTGATAATTCACCTTTAATGAATAATTTCCGTCAATATTTACCTTCCCTC
CCTCAATCGGTTGAATGTCGCCCTTTTGTCTTTGGCGCTGGTAAACCATATGAATTTTCTATTGATTGTGACAAAATAAACTTATT
CCGTGGTGTCTTTGCGTTTCTTTTATATGTTGCCACCTTTATGTATGTATTTTCTACGTTTGCTAACATACTGCGTAATAAGGAGTC
TTAATCATGCCAGTTCTTTTGGGTATTCCGTTATTATTGCGTTTCCTCGGTTTCCTTCTGGTAACTTTGTTCGGCTATCTGCTTACTT
TTCTTAAAAAGGGCTTCGGTAAGATAGCTATTGCTATTTCATTGTTTCTTGCTCTTATTATTGGGCTTAACTCAATTCTTGTGGGTT
ATCTCTCTGATATTAGCGCTCAATTACCCTCTGACTTTGTTCAGGGTGTTCAGTTAATTCTCCCGTCTAATGCGCTTCCCTGTTTTT
ATGTTATTCTCTCTGTAAAGGCTGCTATTTTCATTTTTGACGTTAAACAAAAAATCGTTTCTTATTTGGATTGGGATAAATAATATG
GCTGTTTATTTTGTAACTGGCAAATTAGGCTCTGGAAAGACGCTCGTTAGCGTTGGTAAGATTCAGGATAAAATTGTAGCTGGGT
GCAAAATAGCAACTAATCTTGATTTAAGGCTTCAAAACCTCCCGCAAGTCGGGAGGTTCGCTAAAACGCCTCGCGTTCTTAGAA
TACCGGATAAGCCTTCTATATCTGATTTGCTTGCTATTGGGCGCGGTAATGATTCCTACGATGAAAATAAAAACGGCTTGCTTGTT
CTCGATGAGTGCGGTACTTGGTTTAATACCCGTTCTTGGAATGATAAGGAAAGACAGCCGATTATTGATTGGTTTCTACATGCTC
GTAAATTAGGATGGGATATTATTTTTCTTGTTCAGGACTTATCTATTGTTGATAAACAGGCGCGTTCTGCATTAGCTGAACATGTTG
TTTATTGTCGTCGTCTGGACAGAATTACTTTACCTTTTGTCGGTACTTTATATTCTCTTATTACTGGCTCGAAAATGCCTCTGCCTA
AATTACATGTTGGCGTTGTTAAATATGGCGATTCTCAATTAAGCCCTACTGTTGAGCGTTGGCTTTATACTGGTAAGAATTTGTATA
ACGCATATGATACTAAACAGGCTTTTTCTAGTAATTATGATTCCGGTGTTTATTCTTATTTAACGCCTTATTTATCACACGGTCGGT
ATTTCAAACCATTAAATTTAGGTCAGAAGATGAAATTAACTAAAATATATTTGAAAAAGTTTTCTCGCGTTCTTTGTCTTGCGATT
GGATTTGCATCAGCATTTACATATAGTTATATAACCCAACCTAAGCCGGAGGTTAAAAAGGTAGTCTCTCAGACCTATGATTTTGA
TAAATTCACTATTGACTCTTCTCAGCGTCTTAATCTAAGCTATCGCTATGTTTTCAAGGATTCTAAGGGAAAATTAATTAATAGCG
ACGATTTACAGAAGCAAGGTTATTCACTCACATATATTGATTTATGTACTGTTTCCATTAAAAAAGGTAATTCAAATGAAATTGTT
AAATGTAATTAATTTTGTTTTCTTGATGTTTGTTTCATCATCTTCTTTTGCTCAGGTAATTGAAATGAATAATTCGCCTCTGCGCGA
TTTTGTAACTTGGTATTCAAAGCAATCAGGCGAATCCGTTATTGTTTCTCCCGATGTAAAAGGTACTGTTACTGTATATTCATCTG
ACGTTAAACCTGAAAATCTACGCAATTTCTTTATTTCTGTTTTACGTGCAAATAATTTTGATATGGTAGGTTCTAACCCTTCCATTA
TTCAGAAGTATAATCCAAACAATCAGGATTATATTGATGAATTGCCATCATCTGATAATCAGGAATATGATGATAATTCCGCTCCTT
CTGGTGGTTTCTTTGTTCCGCAAAATGATAATGTTACTCAAACTTTTAAAATTAATAACGTTCGGGCAAAGGATTTAATACGAGTT
GTCGAATTGTTTGTAAAGTCTAATACTTCTAAATCCTCAAATGTATTATCTATTGACGGCTCTAATCTATTAGTTGTTAGTGCTCCT
AAAGATATTTTAGATAACCTTCCTCAATTCCTTTCAACTGTTGATTTGCCAACTGACCAGATATTGATTGAGGGTTTGATATTTGA
GGTTCAGCAAGGTGATGCTTTAGATTTTTCATTTGCTGCTGGCTCTCAGCGTGGCACTGTTGCAGGCGGTGTTAATACTGACCGC
CTCACCTCTGTTTTATCTTCTGCTGGTGGTTCGTTCGGTATTTTTAATGGCGATGTTTTAGGGCTATCAGTTCGCGCATTAAAGAC
TAATAGCCATTCAAAAATATTGTCTGTGCCACGTATTCTTACGCTTTCAGGTCAGAAGGGTTCTATCTCTGTTGGCCAGAATGTCC

CTTTTATTACTGGTCGTGTGACTGGTGAATCTGCCAATGTAAATAATCCATTTCAGACGATTGAGCGTCAAAATGTAGGTATTTCC
ATGAGCGTTTTTCCTGTTGCAATGGCTGGCGGTAATATTGTTCTGGATATTACCAGCAAGGCCGATAGTTTG

References

[1] M. Alhabeb, K. Maleski, B. Anasori, P. Lelyukh, L. Clark, S. Sin, Y. Gogotsi, *Chem. Mater.* **2017**, *29*, 7633.

[2] T. S. Mathis, K. Maleski, A. Goad, A. Sarycheva, M. Anayee, A. C. Foucher, K. Hantanasirisakul, C. E. Shuck, E. A. Stach, Y. Gogotsi, *ACS Nano* **2021**, *15*, 6420

[3] C. J. Zhang, S. Pinilla, N. McEvoy, C. P. Cullen, B. Anasori, E. Long, S.-H. Park, A. Seral-Ascaso, A. Shmeliov, D. Krishnan, C. Morant, X. Liu, G. S. Duesberg, Y. Gogotsi, V. Nicolosi, *Chem. Mater.* **2017**, *29*, 4848.

[4] I. Kaminska, J. Bohlen, S. Rocchetti, F. Selbach, G. P. Acuna, P. Tinnefeld, *Nano Lett.* **2019**, *19*, 4257.
